# scArchon: a scalable benchmarking framework for assessing single-cell perturbation models

**DOI:** 10.1186/s13059-026-04104-z

**Published:** 2026-05-12

**Authors:** Jean Radig, Robin Droit, Daria Doncevic, Albert Li, Duc Thien Bui, Luis Herfurth, Thaddeus Kühn, Carl Herrmann

**Affiliations:** 1 Institute of Pharmacy and Molecular Biotechnology (IPMB), Heidelberg University, Heidelberg, Germany; 2 BioQuant, Heidelberg University, Heidelberg, Germany

## Abstract

**Background:**

The accurate prediction of cellular responses to perturbations, such as drug treatments, remains a pivotal challenge in single-cell transcriptomics. While numerous deep learning tools have been developed for this task, their systematic benchmarking across diverse datasets and performance metrics has been limited.

**Results:**

Here, we present scArchon, a reproducible, modular benchmarking platform built on Snakemake. It is designed to evaluate perturbation response prediction tools in an unbiased and extensible manner. Employing six representative single-cell RNA-seq datasets, we compare leading methods such as scGen, CPA, trVAE, scPRAM, scVIDR, scDisInFact, SCREEN, scPreGAN, and CellOT against baselines. We assess model performance using a composite of statistical and biological metrics. Our analysis reveals heterogeneous performance. While methods like trVAE, scGen, scPRAM, and scVIDR achieve robust results across multiple datasets, other tools occasionally underperform even compared to linear or control baselines. Notably, models with favorable quantitative scores may fail to retain key biological perturbation signatures, underscoring the need for gene-level evaluation.

**Conclusions:**

scArchon provides a unified, extensible foundation for large-scale, standardized benchmarking of perturbation prediction tools, facilitating methodological transparency and accelerating development in this rapidly evolving field. We encourage adoption of scArchon and sharing of containerized tools to drive progress in single-cell perturbation modeling.

**Supplementary Information:**

The online version contains supplementary material available at 10.1186/s13059-026-04104-z.

## Background

Understanding inter- and intra-patient variability is crucial in personalized medicine, as individual differences in gene expression and cellular composition can significantly influence drug responses [1]. Single-cell technologies, such as single-cell RNA sequencing (scRNA-seq), have emerged as powerful tools to dissect this heterogeneity at the individual cell level. By analyzing gene expression variability among different cell types and across individuals, researchers can identify specific cellular subpopulations that may respond differently to molecular perturbations such as therapeutic agents. Integrating single-cell analyses with high-throughput drug screening platforms enables the assessment of drug efficacy and toxicity in diverse cellular contexts, facilitating the development of tailored treatment strategies [2]. For instance, studies have demonstrated that combining scRNA-seq with drug sensitivity profiling [3] can reveal novel prognostic markers and potential therapeutic targets in cancers like liver cancer, thereby enhancing the precision of treatment approaches [4].

**Fig. 1:**
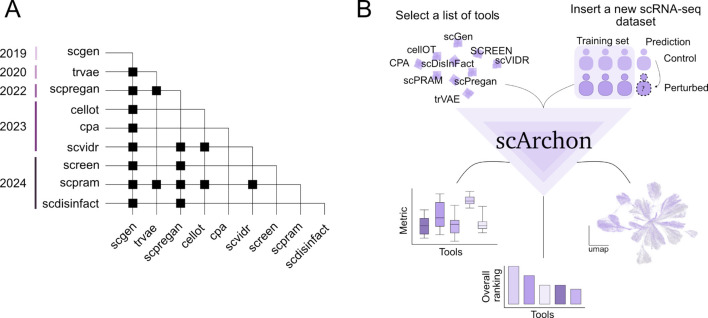
scArchon, a comprehensive benchmarking platform. **A** Matrix indicating benchmarking comparisons among published tools together with their year of publication. Each row represents a tool that has been benchmarked against the tools listed in the columns; a black dot denotes a reported comparison. **B** Overview of the scArchon workflow. scArchon predicts perturbation effects for selected tools on user-provided scRNA-seq datasets. Top right: Input consists of an.h5ad file containing multiple samples in both control and perturbed conditions. Samples can represent different cell types, patients, or species. scArchon uses samples with both control and perturbed cells as a training set to learn perturbation effects, which are then transferred to new control cells (prediction). Top left: Users select the tools to be applied. scArchon executes each tool on the chosen datasets. For every tool, predicted results are saved in.h5ad format alongside the original control and perturbed cells. Bottom left: Beyond generating predicted cells, scArchon performs downstream analyses. When the true perturbation is known, it computes metrics such as MSE, Wasserstein distance, and other biological metrics (see Methods). Bottom right: Visualizations are provided, including dimensionality reduction plots comparing predicted, control, and perturbed cells. Bottom middle: Tools are ranked based on an aggregate performance score

Recent progress in machine learning has introduced new opportunities for in-silico predictions of the transcriptional response of cells subjected to drug treatments or other perturbations [5]. Numerous tools have been developed to predict cellular changes under different stimuli, many claiming to generalize to new, unseen cells (out-of-distribution problem) [6]. However, newer methods often neglect benchmarking against all earlier tools, resulting in fragmented and biased comparisons (Fig. 1A). Moreover, inconsistent use of performance metrics complicates overarching evaluations. So far, no external comprehensive and reproducible benchmark study of perturbation prediction tools has been conducted. Existing benchmarking efforts neither encompass a comprehensive list of tools nor provide unified, reproducible frameworks [7–9].

scArchon (Fig. 1B) is introduced to address these issues. It provides a systematic benchmarking framework for evaluating the latest and most widely used machine learning tools designed to predict perturbation-induced cellular changes. scArchon standardizes performance evaluation across a curated set of datasets, ensures fair comparisons using consistent metrics, and offers fully reproducible pipelines. Notably, scArchon enables users not only to replicate the results presented here but also to easily apply all tools to their own datasets. Unlike previous benchmarking efforts, scArchon resolves common software environment incompatibility issues by providing containerized, scalable solutions that simplify running and fine-tuning models.

We selected nine tools for benchmarking based on their reported ability to predict cellular responses to chemical or bacterial perturbations. Each tool is designed to learn from single-cell RNA-sequencing data that include both control and perturbed conditions across multiple samples. This setup allows the models to capture variability between samples and apply the learned perturbation effects to new control samples. The tools included in our study are: trVAE [10], scDisInFact [11], scVIDR [12], scPRAM [13], scGen [14], SCREEN [15], CPA [16], scPreGAN [17], and CellOT [18]. Importantly, we restricted our analysis to tools that model non-genetic perturbations, as predicting the effects of gene knockouts constitutes a separate problem. Knockout-focused methods, already benchmarked elsewhere [19, 20], typically learn from a single control and multiple knockout conditions to infer unseen perturbations. In contrast, the approaches evaluated here learn the effect of a single perturbation across diverse samples, enabling personalized drug-response predictions for new individuals.

The selected tools differ in their underlying neural network architectures. The most common design uses an encoder to project the single-cell RNA-seq expression matrix into a latent space. Different transformations are then applied to learn the relationship between control and perturbed states. In some models, such as scGen, this transformation is linear. In others, including CellOT, scVIDR, scPRAM, and SCREEN, it is derived using optimal transport. Alternative architectures are also represented: scPreGAN, for instance, employs a generative adversarial network framework to generate perturbed states from control data. This diversity in modeling approaches allows for a systematic evaluation of how different architectures and assumptions impact performance across various biological scenarios.

The tools were evaluated on six publicly available datasets. These include human PBMCs treated with interferon (Kang) [21]; mouse epithelial cells infected with *H. polygyrus* (H. Poly) [22]; mouse liver cells treated with TCDD [22]; cells from multiple species treated with LPS (Species) [23]; glioblastoma cells treated with panobinostat (Glioblastoma) [24, 25]; and human and mouse cells treated with interferon-alpha (Interferon alpha) [26] (Additional Fiel 1: Fig. S1–6). The Kang, H. Poly, Nault, and Species datasets were selected because they are curated, preprocessed, and directly accessible online, reducing variability from data handling and enabling fair model comparisons. Besides, they feature clear and well-defined perturbation effects, making them suitable for baseline evaluations. The glioblastoma dataset was selected for its clinical relevance. It presents a more subtle, clinically realistic challenge, enabling us to assess tool robustness under more variable conditions. The interferon-alpha dataset allows us to evaluate the tools’ ability to transfer learned perturbations from mouse to human, an ability that is clinically important for translational drug research.

By systematically comparing tools on unified tasks and datasets using a variety of metrics, scArchon provides researchers with a clear understanding of each method's strengths and limitations. The automated, extensible pipeline ensures that benchmarking remains reproducible, scalable, and relevant as new tools emerge. Beyond identifying the best-performing models, this work emphasizes the importance of testing methods on realistic, challenging datasets to ensure their practical applicability.

## Results

### Experimental design of the benchmark study

We developed scArchon, a benchmarking platform including nine state-of-the-art tools performing perturbation predictions. To ensure that our comparison is fair towards all tools, we first confirmed that we were able to reproduce the tools’ results with our pipeline (Additional File 2).

We selected six different datasets of varying complexity. We first define the terms that we will use in this benchmark. For all six datasets, models were trained to predict the *perturbed* state (stimulation, infection, or treatment) from the *control* state. Training used all available *covariates*, such as cell types, species, or patients, except one. The held-out covariate was reserved for evaluation. This task is referred to as *out-of-distribution* prediction and tests a model’s ability to generalize to unseen groups. Furthermore, each experiment was repeated in a leave-one-out cross-validation approach iterating over all values of the covariate. We refer to these separate experiments as *prediction folds* throughout the manuscript.

To comprehensively evaluate model performance, we employed complementary approaches: dimensionality reduction visualizations (PCA, t-SNE, UMAP) as well as quantitative metrics (including Mean Squared Error (MSE), Wasserstein distance [27], and t-tests). We also assessed model performance by comparing differentially expressed genes (DEGs) between control and perturbed cells to those between control and predicted cells, measuring their overlap. Additionally, we performed gene set enrichment analysis on the top DEGs to compare enriched biological terms. For the H. Poly, Nault, Kang and Interferon alpha datasets, we used Gene Ontology (GO) terms [28]. For the glioblastoma dataset, we used Molecular Signatures Database (MSigDB) cancer hallmark gene sets [29]. These gene sets are specifically curated to reflect key oncogenic pathways and are more relevant for cancer-related contexts. This allowed us to evaluate whether the models captured both gene-level changes and broader functional patterns.

### Choice of dimensionality reduction method affects interpretation of results

In the original publications of the different tools, performance is often illustrated through visualization in a reduced-dimensional space, aiming to show the alignment between predicted and perturbed cell states. However, the dimensionality reduction techniques used differ between studies, as certain methods may visually suggest stronger overlap than others. We therefore aimed at systematically showcasing all representations for a fairer comparison between tools.

**Fig. 2:**
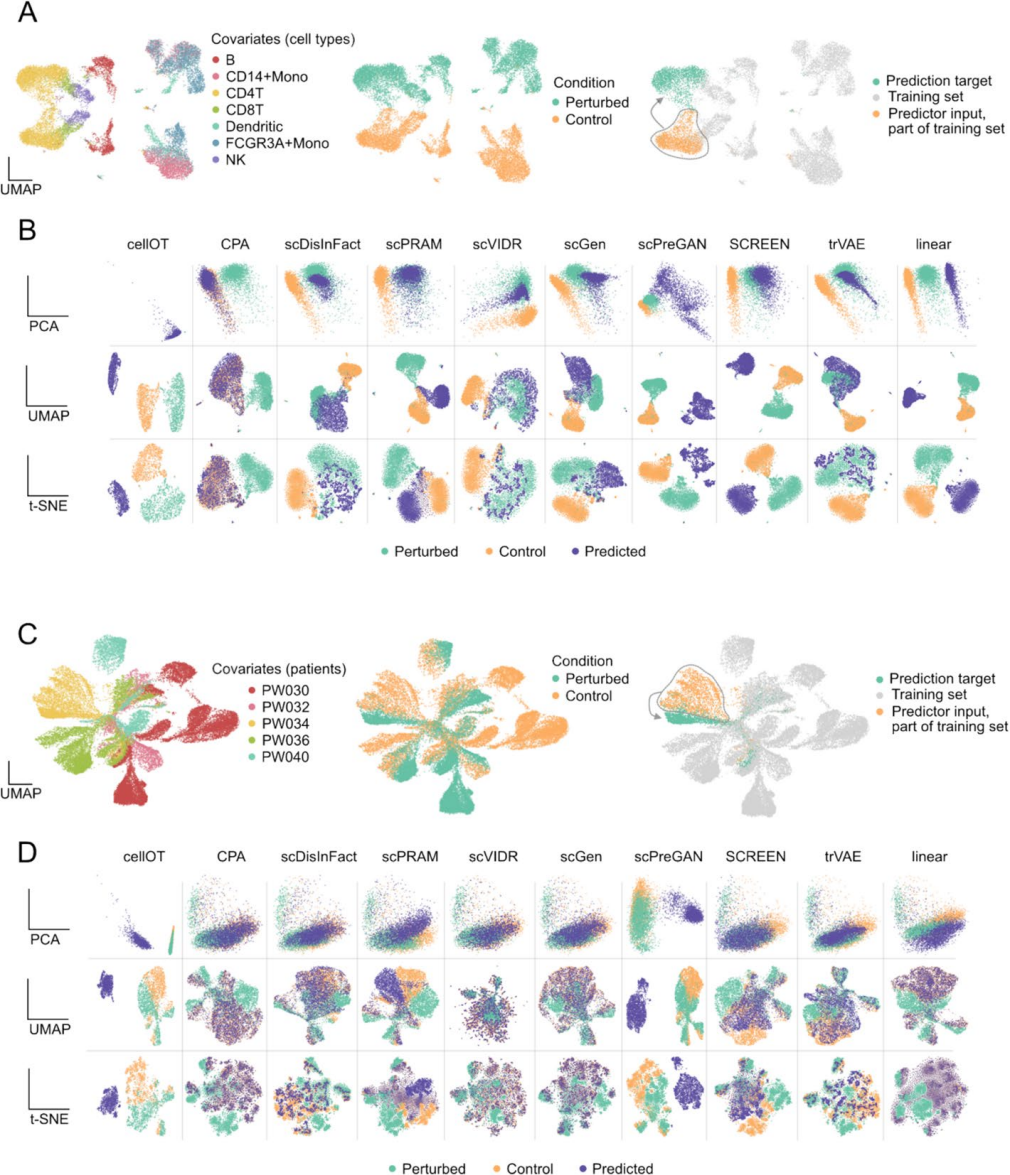
Heterogeneity of the predictions across tools and datasets. The results of CD4T cells from Kang (**A,B**) and the PW034 patient from the Glioblastoma (**C,D**) dataset are shown. **A,C** UMAP representation of the dataset colored by covariate (left), condition (middle), and experimental setup (right). **B,D** PCA, UMAP and t-SNE embeddings of control, perturbed and predicted cells for the different tools

As a primary example, we present the results obtained on the Kang dataset (Fig. 2A–B). This dataset includes seven immune cell types, each with paired control and perturbed conditions. We analyze the prediction fold where models were trained on all cell types except CD4T cells, which were held out as an independent test set (Fig. 2A).

In the PCA representation (Fig. 2B), the overlap between predicted and perturbed cells is most evident for scPRAM, scDisInFact, scGen, scVIDR, and trVAE. This indicates that these models are able to closely approximate the true perturbation response. In contrast, CellOT fails to achieve any overlap. The predicted and perturbed cells are confined to a restricted region of the PCA space. CPA consistently maps predicted cells onto the control distribution rather than the perturbed one. scPreGAN does not produce overlap with either the control or the perturbed cells, which shows a lack of alignment with both reference distributions. The linear model shifts the control cells toward the perturbed state but fails to achieve overlap, which reflects its limited capacity to capture the complexity of the perturbation response.

When examining the UMAP representation, we observe that some methods that appeared to perform well in PCA space show reduced overlap. Notably, scPRAM, which exhibits near-perfect overlap in PCA, shows a clear separation between predicted and perturbed cells in UMAP. A similar trend is observed for scVIDR and SCREEN, where overlap decreases when switching from PCA to UMAP. In contrast, scDisInFact, scGen, and trVAE produce consistent overlaps across both PCA and UMAP. For the remaining models, UMAP often further emphasizes the lack of overlap already seen in PCA. Finally, the t-SNE representation closely mirrors the patterns seen with UMAP and does not provide additional insights beyond those already captured.

The example above highlights the risk of selective visualization. It also underscores the challenge of drawing reliable conclusions from low-dimensional representations. For instance, one might interpret strong alignment between predicted and perturbed cells using PCA, but arrive at the opposite conclusion when viewing the same data through UMAP [30, 31]. Such inconsistencies reveal the inherent subjectivity of visual interpretations based on dimensionality reduction.

To illustrate this on a more challenging dataset, we reproduce these results using the glioblastoma dataset (Fig. 2C) [24, 30]. Here, the objective is to predict the effect of panobinostat treatment on control cells from patient PW034. In Fig. 2D, we observe that even tools that performed well on the Kang dataset tend to produce predicted cells that align more closely with the control group rather than the perturbed group. This pattern suggests a broader limitation in tool generalization when the perturbation effect is masked by confounding effects like inter-patient variability. Results obtained on all experiments for all datasets can be found in Additional File 1: Fig. S5—8.

### Some tools perform no better than a linear model

We quantified tool performance using multiple metrics aggregated over a series of systematic experiments. The results were compared against a *linear model*, which predicts perturbation by adding the mean expression difference between perturbed and control cells to the cells being predicted. The *control baseline* consists simply of the unperturbed cells and represents the absence of any effect.

**Fig. 3:**
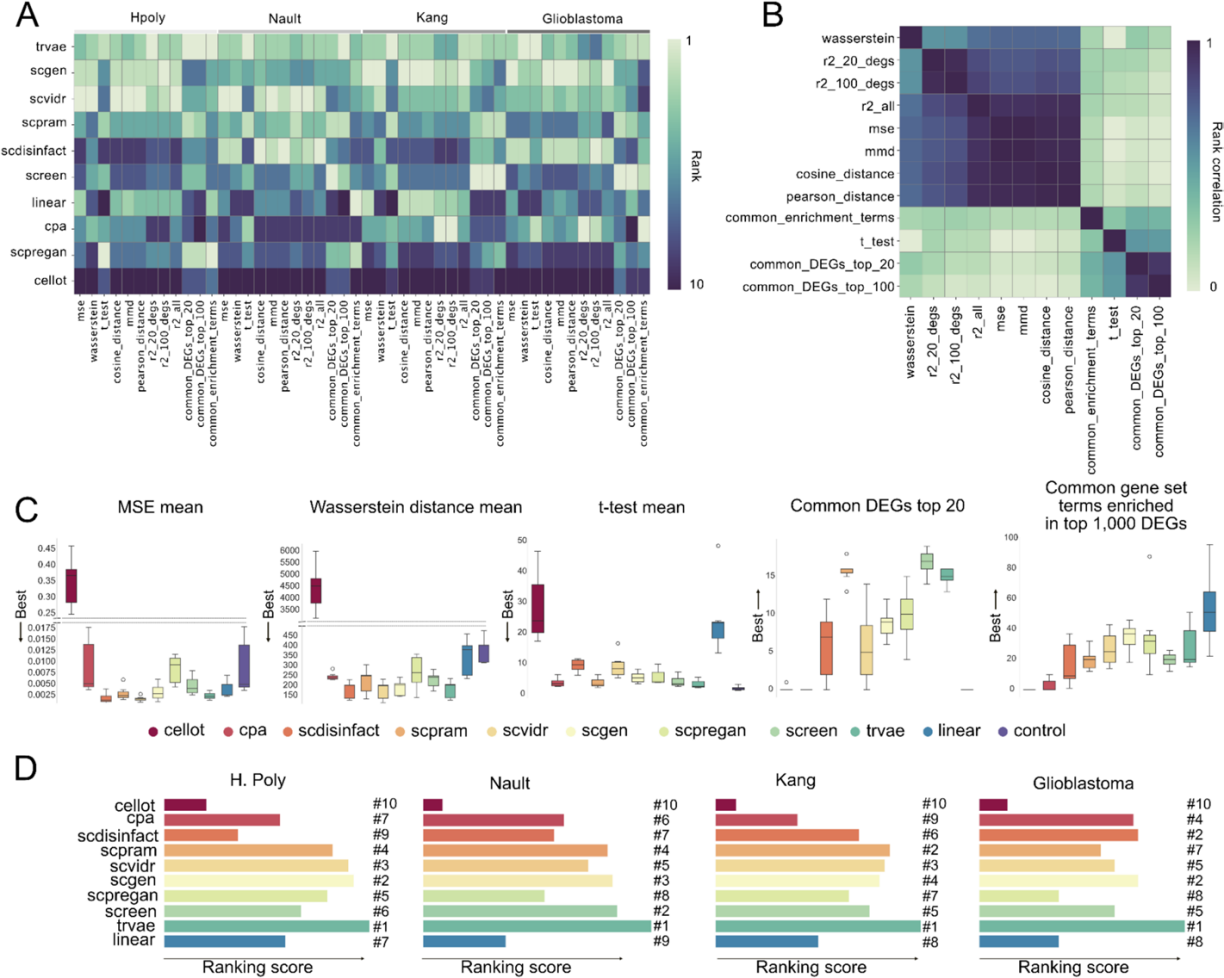
Analysis of scArchon’s output across tools and datasets. **A** Heatmap showing the performance of the different tools across datasets. Each entry represents the ranking of one tool for a particular metric and dataset. The ranking is based on the average score across all prediction folds (cell types, patients or species) for this dataset. The tools are sorted by their overall ranking. By construction, DEG-based metrics cannot be evaluated on the control baseline (crossed out entries). Common enrichment terms could not be computed for the Species dataset because the genes are not mapped to the corresponding annotation database.** B** Correlation between tool rankings across different metrics, showing the similarity between different metrics. **C** Boxplots showing five evaluation metrics computed on the Kang dataset. Each box represents the mean result over the prediction folds.** D** Overall ranking of each tool across datasets. x-axis represents an ad-hoc ranking score (see Methods). The Species dataset was excluded, as common gene set enrichment could not be computed for this dataset

Tools were ranked according to their average performance across prediction folds for each dataset. The highest mean score for a given metric and dataset received the top rank, with other tools ranked sequentially. These rankings, illustrating each tool’s relative position within individual datasets, are shown in Fig. 3A. The top performing tools in the overall ranking appear to recapitulate the visual inspection from the previous section. Looking at individual datasets, we observe heterogeneity in the performance of the tools, for instance scDisInFact performing poorly on H. Poly or Kang datasets but well on Nault and Glioblastoma datasets.

Consistent with [32], we observed that some metrics produced similar tool rankings (Fig. 3A). By definition, some metrics are mathematically related, such as MSE and R2. Indeed, looking at the correlation of the rankings produced by the different metrics, we observe high correlation on some aggregate metrics (Fig. 3B). To generate an overall ranking across datasets and avoid ranking biases, we selected a subset of metrics that capture distinct aspects of performance. These metrics are sufficiently uncorrelated, preventing bias toward tools that excel in only a narrow set of criteria. Using all metrics despite their high correlation could unfairly favor tools optimized for specific measures [7].

After analyzing metric correlations, we selected the following five for our composite evaluation: R2 on the top 2000 highly variable genes (HVGs), Wasserstein distance, t-test score, overlap in top 20 DEGs, and overlap in enriched gene sets derived from the top 1,000 DEGs. This combination balances statistical accuracy (Wasserstein metric, t-test score) with biological interpretability (top HVGs, top DEGs, enriched terms) while reducing redundancy. Note that common gene set enrichment could not be computed for the Species dataset because the genes are not mapped to the corresponding annotation database.

To illustrate tool performance, we analysed results on all datasets across the selected metrics (Fig. 3C for Kang and Additional File 1: Fig. S7—11 for the others). On the Kang dataset, certain tools consistently ranked high on measures such as R2 on the top 2000 highly variable genes, Wasserstein distance, and t-test statistics. Notably, scVIDR, scPRAM, scGen, scDisInFact, and trVAE were among the top performers with low variability across the prediction folds. In contrast, CellOT consistently showed the poorest performance, with CPA and SCREEN also ranking low. Importantly, several tools performed worse than both the linear model and the control baseline. For example, in the Kang dataset, no tool outperformed the control on the t-test metric. For other metrics as well, the predictions deviated further from the true perturbed state than simply using the unaltered control or applying a constant mean shift. This suggests that some models may generate noisy or biologically implausible predictions.

Interestingly, evaluation based on common DEGs and enriched biological terms revealed that some tools with lower quantitative metric scores still captured meaningful biological signals. For example, SCREEN and scPreGAN ranked among the top performers in these biological assessments. The reverse was also true. Tools like CPA and scDisInFact had fair quantitative metric values but poor overlap at the level of DEGs. These results highlight a key limitation. Models that appear convincing in aggregate metrics can fail to preserve the underlying gene-level perturbation structure. This underscores the importance of combining rigorous gene-level quantitative evaluations to accurately interpret and apply perturbation prediction models. Results obtained on the other datasets can be found in Additional File 1, Fig. S6B-8B.

To provide a high-level comparison of tool performance across datasets, we computed a unified ranking based on the five key evaluation metrics shown in Fig. 3C. We normalized rankings into a single composite score ranging from 0 to 1. A score of 1 indicates a tool that consistently ranked first across all experiments and metrics, while a score of 0 indicates a tool that consistently ranked last.

This ranking approach was applied across Kang, H. Poly, Nault and Glioblastoma datasets in the benchmark. The Species dataset was excluded because gene set enrichment could not be computed. The results are summarized in Fig. 3D. We find that trVAE is the top-performing method overall. Interestingly, scDisInFact, which ranks relatively low on the Kang (6th) and H. poly (9th) datasets, achieves second place on the glioblastoma dataset, a task which is arguably more complex. The linear model ranks twice in 8th place, and once in 7th. Notably, tools such as CellOT, CPA and scPreGAN tend to sometimes fall below the linear model in performance. In contrast, methods like scGen, scPRAM, and scVIDR consistently rank near the top, demonstrating more robust performance across different biological contexts.

### Deeper analysis of biological metrics reveals hallucinations of the prediction models

**Fig. 4:**
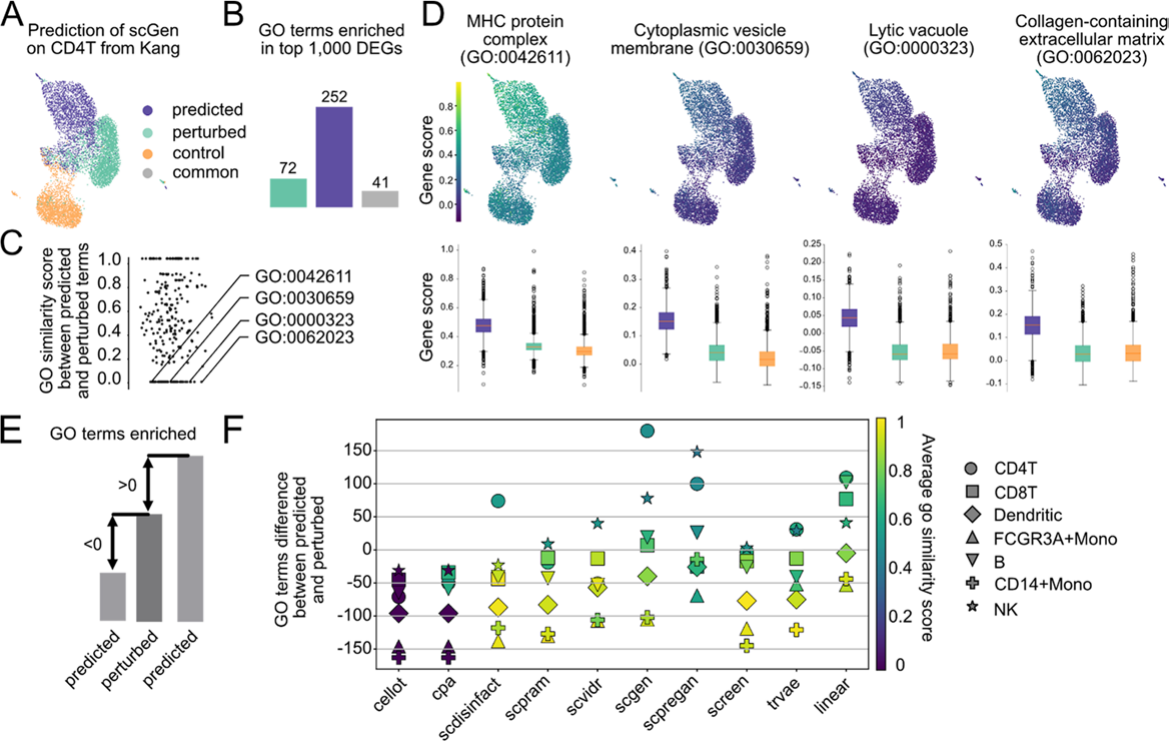
Deeper analysis of biological metrics reveals hallucination of prediction methods. **A** UMAP showing the predicted expression profile alongside control and perturbed cells.** B** Number of Gene Ontology (GO) terms enriched among the top 1,000 differentially expressed genes between control and predicted (predicted terms, blue) or control and perturbed cells (baseline terms, green), with grey indicating the shared enriched terms. **C** Dotplot showing the highest semantic similarity score for each predicted term compared to all baseline terms. Four predicted GO terms with a maximum similarity score of zero were arbitrarily selected for deeper analysis.** D** Signature scores based on average gene expression for the four selected GO terms with zero similarity. Boxplots show the score differences between predicted and perturbed cells, all of which are statistically significant (Mann–Whitney U test, *p* < 0.01). **E** Computation schematic. We compare the number of predicted terms with the number of baseline terms. The difference is positive when more GO terms are predicted than in the reference, and negative when fewer are predicted. **F** Results across experiments. Each dot represents the prediction fold (celltype), the Y axis represents the difference between the number of predicted and baseline terms. The color indicates the average semantic similarity between predicted and baseline terms

A closer inspection of the results in Fig. 3C (right panel) reveals that the linear model achieved the highest overlap in enriched GO terms with the perturbed condition on the Kang dataset, with an average of 54 shared terms over the prediction folds. This was followed by scPreGAN and scGen, with 36 and 35 overlapping terms, respectively, while trVAE and scVIDR also performed well, with 29 and 27 shared terms. These values must be interpreted with caution, as the number of shared terms is inherently influenced by the total number of predicted enriched terms (referred to as predicted terms). Tools that produce a large number of predicted terms may inflate overlap, increasing the risk of false positives. For instance, although scGen shows strong performance both qualitatively and across multiple metrics (Figs. 2B and 3A–D), it predicted 252 enriched GO terms for CD4 T cells, compared to only 72 in the perturbed condition (referred to as baseline terms) (Fig. 4A-B). Of these, 41 terms overlapped with the baseline terms.

To evaluate whether the additional predicted terms were biologically meaningful, we computed semantic similarity scores between predicted terms and baseline terms (Fig. 4C). As expected, the common terms had a similarity score of 1. Several predicted-only terms had a similarity score of 0 to all baseline terms. To investigate whether these might represent false positives, we arbitrarily selected four such terms and examined the expression of the genes associated with them across control, predicted, and perturbed conditions (Fig. 4D). In each case, the expression of the associated genes was significantly higher in the predicted condition than in the perturbed one (based on a Mann–Whitney U test). This suggests that these predictions might result from artificially elevated gene expression. It can potentially lead to spurious GO term enrichment and misleading downstream biological conclusions.

We performed this analysis on the Kang dataset prediction folds, comparing the number of predicted terms by each method to the number of baseline terms (Fig. 4E–F). Some tools, such as scGen and scPreGAN, tend to predict more enriched terms than observed in the perturbed condition, while others consistently yield fewer. Interestingly, tools that predict fewer terms than the ground truth often achieve higher average semantic similarity scores—indicating fewer, but more biologically relevant predictions (for example SCREEN on monocytes). However, scGen and scPreGAN do not always over-predict. As shown in Additional File 1, Fig. S9 for the H. poly dataset (also evaluated using GO similarity), these tools often predict a similar or even smaller number of terms compared to others.

These findings highlight that biological evaluation of prediction results can be challenging, as tools may produce misleading biological enrichments. We believe that this is similar to the known phenomenon of hallucinations in LLMs. High overlap in enriched terms or DEGs does not always reflect true biological relevance, especially when driven by inflated or unstable predictions. This underlines the need for careful interpretation and complementary analyses when assessing the biological fidelity of perturbation models.

### Cross-species translation proves to be a challenge beyond the reach of current tools

We evaluated whether the tools can transfer learned perturbation effects across species. To investigate this, we first used the Species dataset, which contains cells from four organisms (mouse, rabbit, pig, and rat) each treated with lipopolysaccharide (LPS) for six hours (Additional File 1: Fig. S3). In this setting, the models were trained to learn the LPS response in three species and then predict the response in the held-out fourth species. However, performance on these cross-species folds indicated that the tools were unable to produce meaningful overlap in low-dimensional embeddings, and the linear model ranked among the top performers in terms of MSE. These findings motivated us to explore whether cross-species transfer might be more feasible in a biologically closer context, specifically from mouse to human.

**Fig. 5:**
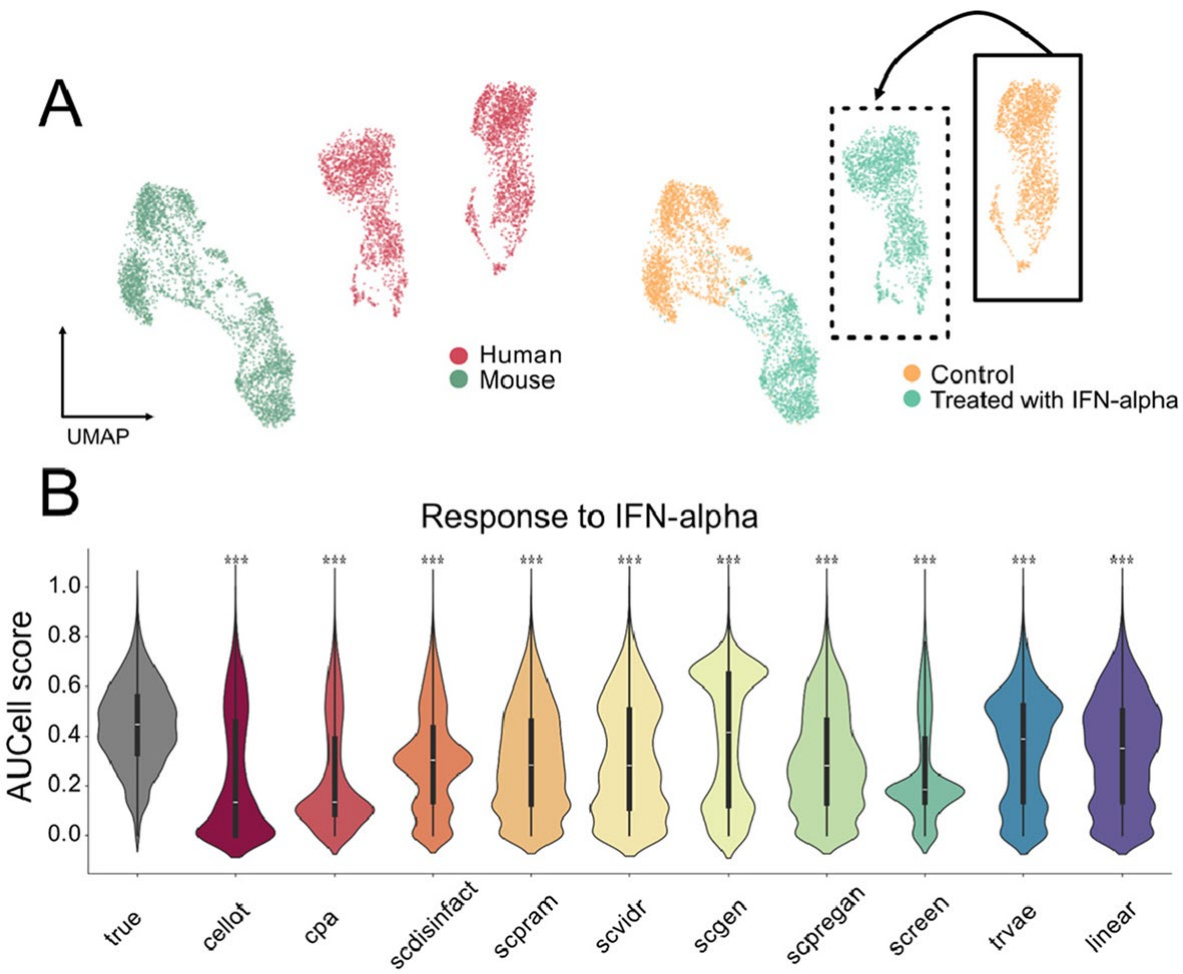
Models struggle to transfer learned perturbations from mouse to human. **A** Overview of the interferon-alpha dataset and the cross-species prediction task. Left: cells colored by species. Right: cells colored by condition (control vs. interferon-alpha treated). The arrow indicates the experimental setup, in which human control cells are used as input and models attempt to predict their perturbed state.** B** Distribution of AUCell scores for the interferon-α response pathway (GO:0035455) across all tools. Model-predicted scores are compared with those from true perturbed human cells using a two-sided Kolmogorov–Smirnov test. Tools with significantly different distributions are indicated with stars above the violins (***p < 0.001)

To this end, we used the interferon-alpha dataset, which includes both mouse and human T cells exposed to interferon-alpha (Additional File 1: Fig. S6). Here, too, the results were mixed, and the models again failed to generate substantial alignment between species in reduced-dimensional space (Additional File 1: Fig. S12). To further examine whether the predicted perturbations captured the underlying biology of the interferon response, we quantified the activity of the GO: Response to Interferon Alpha gene program using AUCell scores. This approach bypasses single-gene noise and evaluates whether a coordinated transcriptional module is correctly recovered. Across all tools, the distribution of AUCell scores differed significantly from that of the true perturbed human cells (p < 0.001, two-sided KS-test), demonstrating that the models did not faithfully reproduce the pathway-level activation induced by interferon-alpha (Fig. 5). This gene-set–level discrepancy reveals that even when individual gene predictions appear reasonable, the higher-order regulatory structure of the perturbation is not preserved across species.

### Tools have different robustness in ablation experiments

**Fig. 6:**
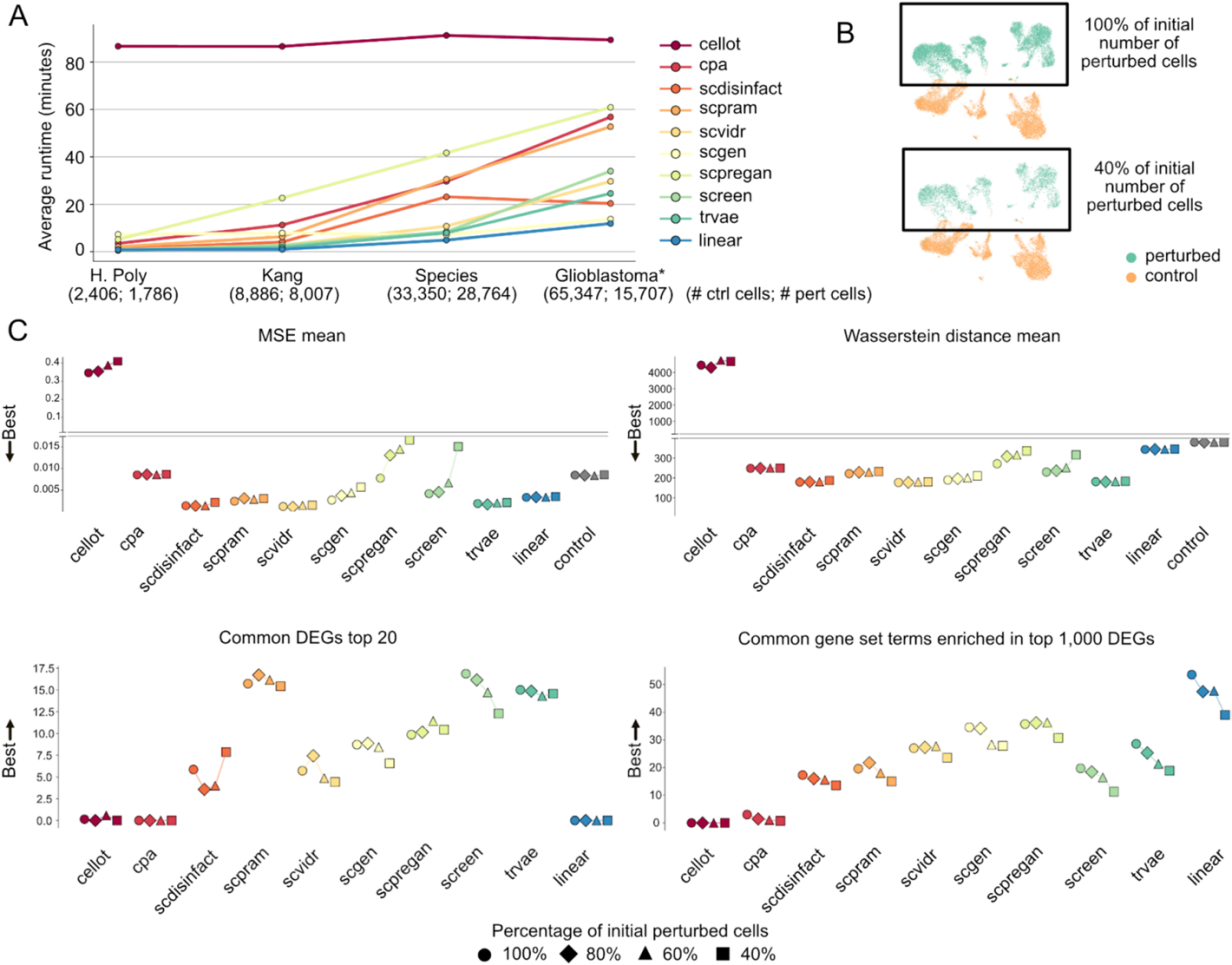
Runtime and ablation experiment performance of tools highlights substantial differences between methods. **A** Runtime of each tool. Glioblastoma* refers to the dataset prior to balancing the number of perturbed and control cells. This distinction is used to illustrate how runtime scales with increasing cell counts.** B** Ablation experiment design: four datasets were generated from the Kang dataset by progressively reducing the number of initially perturbed cells—100%, 80%, 60%, and 40%.** C** Performance metrics across these datasets. Top left: Mean Squared Error (MSE). Top right: Wasserstein distance. Bottom left: number of differentially expressed genes (DEGs) shared between predicted and perturbed cells. Bottom right: number of enriched gene sets among the top 1,000 DEGs between predicted and perturbed cells

In addition to evaluating model performance, we examined tool efficiency and robustness through runtime and ablation studies. Figure 6A shows the average runtime per prediction fold across datasets of increasing cell numbers. All tools scale with dataset size except CellOT, which maintains a constant—and highest overall—computation time. To test robustness, we conducted an ablation experiment by progressively reducing the number of perturbed cells to 80%, 60%, and 40% (Fig. 6B). Most tools remained stable on MSE and Wasserstein scores, with slight worsening of performance observed for SCREEN, scPreGAN, and CellOT (Fig. 6C). For DEG overlap, scDisInFact showed minor fluctuations but remained consistent, and similar patterns were seen across other tools. A general decline in the number of enriched terms was noted with fewer perturbed cells.

We further evaluated model sensitivity to gene set size (Additional File 1: Fig. S14A) by progressively reducing the glioblastoma dataset from all genes to 15,000 and 7,000 highly variable genes (HVGs). As shown in Additional File 1: Fig. S14B, runtimes remained constant. For scVIDR and trVAE, reducing the gene set size led to fewer predicted enriched terms, though the top 1000 DEGs were still kept for enrichment analysis. In contrast, Wasserstein distance improved for all tools as the gene set was reduced. Meanwhile, the overlap of the top 20 DEGs increased, and MSE remained stable, suggesting that errors are largely driven by poorly predicted HVGs, while removing less variable genes has minimal impact on performance.

These results show that while most tools are computationally efficient and robust to moderate reductions in data, performance can vary depending on the nature of the ablation. Notably, reducing input genes or stimulated cells affects biological signal recovery more than standard metrics like MSE, highlighting the importance of evaluating models across multiple dimensions, especially biological relevance.

### Working principle of scArchon to evaluate all the tools on a new dataset

The results above highlight the importance of an open, comprehensive, and fair benchmarking framework that incorporates visual, quantitative, and biological evaluations. As the number of tools for perturbation prediction is constantly growing, we developed scArchon, an accessible platform designed to benchmark models across key performance criteria (https://github.com/hdsu-bioquant/scArchon). Crucially, scArchon eliminates the burden of environment setup by using pre-configured Docker images, enabling seamless integration and execution of existing tools. Running scArchon requires an NVIDIA GPU driver that is compatible with CUDA 12.4 or later.

Users can select a subset of perturbation prediction tools to benchmark. Given an input dataset, scArchon executes the full pipeline, encompassing model training, prediction, and evaluation. The outputs include: (1) predicted expression matrices in.h5ad format, (2) evaluation metrics in.csv format and (3) a collection of visualizations.

The framework is modular and customizable. Users can adjust individual tool pipelines, including parameter settings, to better fit their specific experimental design. However, we noted that increasing the number of training epochs generally does not yield significant performance improvements, despite substantially longer runtimes. In line with common practice, most published tools rely on default or fixed parameters across datasets. As such, the parameters used in scArchon serve as a reasonable and fair baseline for initial evaluations.

For more granular or isolated evaluations, users also have the option to run any individual tool independently using its publicly available Docker image.

## Discussion

Out-of-distribution prediction represents a significant challenge. It holds the promise to model cell-specific responses to molecular perturbations such as drug treatment or the effect of infection. If successful, this would represent a groundbreaking advance in the field of personalized medicine. It is therefore especially relevant to evaluate to what extent the tools that have been developed in recent years hold this promise [6]. However, evaluating the quality of these predictions represents an equally complex task, which we have tried to address in this work. Previous similar but more limited efforts have been conducted for example as part of the Open Problems in Single-cell Analysis in a collaborative benchmark at NeurIPS 2023, or the Therapeutics Data Commons effort [33]. Defining the quality of the predictions is heavily influenced by the choice of qualitative and quantitative evaluation metrics. For example, as we have shown, different dimensionality reduction methods provide sometimes contradicting interpretations. These findings indicate that dimensionality reduction visualizations alone cannot serve as reliable criteria for evaluating tool performance. Quantitative metrics are needed. The tools under consideration typically assess their performance based on machine-learning related metrics, such as MSE. According to such metrics, almost half of the tools perform on par or worse than the linear baseline model, in which the perturbation is modelled as a simple constant effect over all cells, highlighting the current limitations in capturing complex perturbation responses. Several of the metrics which are commonly used are mathematically related and do not represent independent evaluation criteria. Scoring methods based on these metrics might lead to a biased evaluation.

Most metrics take into account more global patterns (e.g. distributional profiles) and might not detect if a tool fails to model relevant biological signals. For this reason, in contrast to previous benchmarks, we also included biological gene-level metrics in our study, such as the number of DEGs or the enriched biological terms amongst the top 1000 DEGs that are shared between perturbed and predicted cells [7, 8]. Interestingly, evaluating tools based on these more biological criteria sometimes leads to vastly different rankings compared to the typical quantitative metrics. Some tools perform well on global metrics, but detect enriched biological terms that are not related to the underlying biology. The correlation between the rankings of the quantitative metrics and the biological metrics is low, emphasizing this discrepancy (< 0.5, Fig. 3C). We believe that this is similar to the hallucination effects that are common in complex deep learning approaches such as LLMs and computer vision [34]. Unlike hallucinations in LLMs which generate plausible but false text, biological hallucinations in perturbation models manifest as spurious pathway enrichments that could mislead downstream biological interpretation and experimental design. Including biological priors into the prediction models might alleviate these effects, as they would constrain the predictions to biological plausible solutions [35–37]. These observations underscore the necessity for novel evaluation approaches that better represent the capacity of perturbation prediction tools to model biological responses.

The performance of each tool is highly dependent on the type and strength of the perturbation and varies considerably across testing scenarios. To capture this variability, we evaluated the tools across a broad range of data types and experimental conditions. This approach allows us to assess not only overall performance but also the generalizability of each tool. Such diversity in testing is essential because many tools perform well when the perturbation is the main source of variance but struggle when other confounding effects, such as patient-specific responses, dominate.

Across the metrics used in this study, scVIDR, scPRAM, scGen, scDisInFact, and trVAE consistently outperformed other methods. However, even these top-performing tools show limited accuracy in more challenging datasets. This suggests that predicting the full transcriptional response of each cell may be an overly ambitious and sometimes unnecessary goal. Alternatively, predicting the changes in the activities of specific pathways of interest or the drug sensitivity of cells appears as a more realistic goal which could be sufficient in many application scenarios [38–41].

Our study, because it provides a generic, dataset agnostic benchmarking platform, has limitations. More in-depth biological analyses could be performed beyond the evaluations presented here. In particular, thorough validation of model predictions may, in some cases, require targeted examination of specific marker genes relevant to the dataset under consideration. Such gene-level effects may be obscured when relying solely on aggregate metrics computed across all genes and cells. As an illustration of a possible datasets specific follow-up analysis, for the Kang dataset we examined the expression of selected genes known to respond to interferon-beta treatment (ISGs) (Additional File 1: Fig. S19–28). However, conducting such dataset-specific and biologically informed analyses in a systematic manner would require prior knowledge of the underlying biological system and is therefore difficult to generalize across datasets within the context of a benchmarking pipeline. Nevertheless, because our pipeline outputs the predicted expression profiles for all methods, users can readily perform downstream, data-specific analyses tailored to their biological questions of interest.

Large language models (LLMs) have entered the field of single-cell perturbation modeling. While current implementations primarily focus on perturb-seq–type datasets, we anticipate that LLM-based approaches will increasingly be applied to the broader range of perturbation prediction problems explored in this benchmarking study. For example, the recently developed c2s [42] tool represents an early attempt to tackle these tasks. We evaluated its performance, but it did not outperform the other methods (Additional File 1: Fig. S15—18). Due to memory limitations, we were unable to run the highest-capacity model. Furthermore, the model runtime is prohibitive for large datasets, as the prediction time is directly linked to the number of cells. Due to these limitations, we did not include c2s in the main benchmarking to ensure a fair comparison. Nonetheless, as these models continue to evolve, improvements in accuracy and generalization are expected, making them promising candidates for future perturbation prediction applications.

As the number of tools for predicting perturbations in single-cell datasets continues to grow, we propose the scArchon framework. Built on Snakemake (Mölder et al., 2021), scArchon enables efficient and reproducible testing of perturbation prediction tools. It supports evaluations across diverse datasets using multiple metrics. Its modular design allows for seamless integration of new tools and datasets, promoting large-scale, standardized evaluations. Such a common benchmarking system would not only facilitate transparency in reporting but also accelerate methodological improvements by providing a clear reference for performance assessment [43]. scArchon contributes to this vision by offering a unified platform that enables systematic, reproducible, and transparent evaluation of perturbation prediction tools. As the field progresses, the availability of such a framework could serve as a foundation for measuring improvements in prediction accuracy and biological relevance, fostering innovation and guiding the development of more robust models. Therefore, we encourage the authors of future methods to make use of this unbiased evaluation platform and provide dockerized images of their tools.

## Conclusion

In conclusion, our study highlights the persistent challenges in accurately predicting out-of-distribution cellular responses to perturbations. We demonstrate that commonly used evaluation strategies, particularly those relying on global quantitative metrics, can provide incomplete or misleading assessments of model performance. By incorporating biological gene-level metrics, we reveal substantial discrepancies that underscore the risk of biologically implausible predictions. Even top-performing models show limited robustness across diverse datasets and perturbation contexts, suggesting that current approaches struggle to capture complex biological variability. These findings indicate that predicting full transcriptional responses may not always be a realistic objective, and that focusing on pathway-level or functional outputs may offer more practical alternatives. Furthermore, our results emphasize the need for improved evaluation frameworks that better align with biological relevance. While emerging approaches such as LLM-based models show promise, they are not yet competitive in this setting. Overall, advancing this field will require both methodological innovation and more rigorous, biologically informed evaluation practices.

## Methods

### Datasets

#### Kang dataset

The Kang dataset (Additional File 1: Fig. S1) originates from human peripheral blood mononuclear cells (PBMCs) comprising seven different immune cell types and is publicly available through the GEO repository under accession number GSE96583 [21]. For our work, we used the pre-processed and annotated version provided by [14], which can be downloaded from https://www.dropbox.com/s/wk5zewf2g1oat69/train_pbmc.h5ad?dl=1. The dataset includes 16,893 cells in total, 8,007 control cells and 8,886 interferon-β perturbed cells, and covers 6,998 genes.

#### H. Poly dataset

The H. Poly dataset [44] (Additional File 1: Fig. S2) consists of intestinal epithelial cells from mice that were infected with the parasitic helminth *Heligmosomoides polygyrus* over a ten-day period. The dataset is available as raw data under GEO accession number GSE92332. We used the processed and annotated version published by [14], which can be accessed at https://www.dropbox.com/s/7ngt0hv21hl2exn/train_hpoly.h5ad?dl=1. We retained only cell types with more than 400 cells to ensure statistical robustness and avoid biases due to small sample sizes. Thus, the final dataset contains 2,406 control cells and 1,786 perturbed cells from five different cell types, and 7,000 genes.

#### Species dataset

The species dataset [23] (Additional File 1: Fig. S3) (accession id E-MTAB-6754) consists of cells from the four species mouse, rabbit, pig, and rat treated with lipopolysaccharide (LPS) for six hours. The processed data was downloaded from https://www.dropbox.com/s/eprgwhd98c9quiq/train_species.h5ad?dl=1 which was published by [14]. The dataset consists of 62,114 cells (33,350 control and 28,764 perturbed) and 6,619 genes.

#### Glioblastoma dataset

The Glioblastoma dataset (Additional File 1: Fig. S4) contains single-cell transcriptomic profiles of glioblastoma patient-derived cells and is available as raw data under GEO accession number GSE148842. We used the annotated version by [25], which can be downloaded from https://drive.google.com/file/d/18-KInmm43wKdBX95Gq9xbuzAQwtLjgE9/view?usp=sharing. In our analysis, we subset the dataset to include only five patients (PW030, PW032, PW034, PW036, and PW040) that had both control and panobinostat-treated conditions available, excluding all other treatment groups. When duplicated gene names are present, we keep the counts of the first instance. Counts were normalised to 10,000 and log1p. The original dataset consists of 81,054 cells, of which 65,347 are control and 15,707 are perturbed, and 20,098 genes. However, we randomly downsampled the control cells of the dataset to match the number of perturbed cells.

#### Nault dataset

The Nault dataset [22] (Additional File 1: Fig. S5) original data can be accessed using GEO accession number GSE184506. The raw gene expression matrix was taken from the GitHub repository (https://github.com/BhattacharyaLab/scVIDR) and processed using the scanpy toolkit. Low-quality cells expressing fewer than 200 genes were removed, and genes detected in fewer than 10 cells were excluded from the analysis. The filtered data was normalised by total counts per cell to 10′000 and transformed using natural logarithm with a pseudo count to 1. We filtered for highly variable genes. The final dataset contains 30,927 cells and 6,999 genes.

#### Interferon alpha dataset

We used the data presented in the paper [26] where human blood and mouse spleen cells were exposed to interferon alpha. The data is available under accession code GSE142637. We have downloaded the datasets GSM4233280 (human control), GSM4233278 (human interferon alpha), GSM4233283 (mouse control) and GSM4233281 (mouse interferon alpha). We mapped mouse genes to human genes. Only genes present in both sets were kept. We concatenated the datasets, selected cells for the cells who had at least 200 expressed genes, less than 10% of mitochondrial reads and a minimal number of 1000 counts per cell. We filtered out genes that were present in less than 3 cells. We normalised the counts to 10,000 and performed log1p transformation. We annotated the cells using celltypist using the model “Immune_All_High”. We obtained similar cell annotations to those obtained in the original paper. We needed to select cell types that are present in both species in sufficient and comparable amounts. The only cell type that satisfied this requirement were the T-cells. We therefore subsetted the dataset for only the T-cells. The final dataset consists of 6,964 cells and 12,542 genes and is presented hereafter.

### Model selection

#### Task definition

The perturbation task is defined as predicting the transcriptional response of a cell to a specific experimental perturbation, such as a drug or stimulation. The model learns the effect of a single perturbation from a set of control–perturbation pairs across multiple samples. Given a new sample with different biological characteristics (e.g., a different cell type or a different patient), the model transfers the learned perturbation to predict the perturbed state of that sample. This formulation allows evaluation of how well models capture and generalize perturbation effects across diverse biological contexts.

#### Benchmarked tools

Each of the selected methods employs deep learning and relies on dimensionality reduction to project high-dimensional gene expression data into a latent representation where it then learns a mapping from control to perturbed state.

scGen (https://github.com/theislab/scgen, commit d79e1f0, version 2.1.0) uses a variational autoencoder (VAE) framework in which the perturbation effect is modeled as a simple linear vector shift in the latent space that can then be applied to new control samples to simulate the perturbed state.

CellOT (https://github.com/bunnech/cellot, commit 522d2b9), scPRAM (https://github.com/jiang-q19/scPRAM, commit a462429), scVIDR (https://github.com/BhattacharyaLab/scVIDR, commit 605825f), and SCREEN (https://github.com/Califorya/SCREEN, commit c0dc236) use optimal transport (OT) in the latent space to learn a transport map that shifts the distribution of control cells toward that of perturbed cells.

CPA (Compositional Perturbation Autoencoder) (https://github.com/theislab/cpa, commit fbd7c02) disentangles confounding variables—such as cell type, patient, or batch—from the perturbation effect in the latent space by separately learning them through embedding layers. It makes the assumption that the latent space can then be described as the sum of the different components. We modified the model following guidance found in the CPA GitHub repository (Additional File 2).

scDisInFact (https://github.com/ZhangLabGT/scDisInFact, commit 9466ecf) also learns a disentangled latent space that separates batch effects from condition-specific signals. It addresses three tasks simultaneously: batch effect removal, identification of condition-associated key genes, and perturbation prediction. This design makes scDisInFact applicable to studies with multiple batches and experimental conditions, where integrated analysis is required.

trVAE (transfer variational autoencoder) (https://github.com/theislab/trVAE_reproducibility, commit 2a594b6) extends the CVAE architecture by modeling the perturbation effect as a condition vector, which is disentangled from batch variables such as cell state, donor, or species. These vectors are integrated in the decoder and optimized using a maximum mean discrepancy (MMD) loss, which aligns the latent representations across different conditions.

scPreGAN (https://github.com/XiajieWei/scPreGAN, commit d9fa8ea) employs a generative adversarial network (GAN) architecture, combining it with an encoder to learn a generative mapping from control to perturbed states. The GAN framework encourages the model to produce perturbed cell states that are both realistic and aligned with the underlying biological signal, using adversarial training to improve fidelity.

c2s (https://github.com/vandijklab/cell2sentence, commit a6efaf0) reformulates single-cell expression profiles into a natural-language representation by converting each cell into a sequence of gene names ordered by decreasing expression. This “cell sentence’’ format enables large language models (LLMs) to operate directly on scRNA-seq data using standard language modeling architectures. The method provides multiple pre-trained models, with the newer versions benefiting from larger training corpora and improved generalization. In our setting, memory constraints prevented the use of these larger models. We therefore relied on the lighter pythia-160 m-c2s model and reduced the batch size from two to one to ensure stable execution, while keeping all other parameters at their default values.

Unless otherwise noted, all tools were executed using their default parameters as provided in the specified GitHub commit.

#### Linear model baseline

For each gene, we compute the average expression across all cells in the control and perturbed conditions within the training set. The difference between these two averages represents the estimated perturbation effect for that gene. To generate predictions on the test set, we add this gene-wise difference to the expression profile of each test cell from the control condition.

#### Control baseline

We used the control condition directly by applying the same metrics to the unmodified control cells compared to the true perturbed cells. For metrics that rely on differential comparisons, such as those requiring DEG analysis between control and perturbed states, scores for the control baseline could not be computed.

### Evaluation metrics

#### Dimensionality reduction techniques

Principal component analysis was performed using the function scanpy.pp.pca [45] with the number of components set to 50 and random_state set to 42. UMAP was computed using scanpy.tl.umap with min_dist set to 0.5, spread set to 1, and random_state set to 42. t-SNE was applied using scanpy.tl.tsne with random_state set to 42.

#### Metrics

MSE, MMD, Wasserstein distance, t-test score, pearson distance and cosine distance were computed using the pertpy.tl.Distance function from pertpy [46]. R^2^ was computed using the implementation from scButterfly [47] (https://github.com/BioX-NKU/scButterfly, commit eb31e04). We calculated R^2^ across three gene subsets: the top 20 DEGs, the top 100 DEGs, the top 2000 highly variable genes and the entire set of genes. Highly variable genes were computed using the scanpy function scanpy.pp.highly_variable_genes with flavor seurat.

#### Ranking methodology and tool score

The tools were ranked based on the average ranks across five metrics: MSE, Wasserstein distance, t-test, common DEGs and common enriched terms. Since the average rank was not intuitive to rank the different tools (the smallest value was representing the best tool), we reversed the score by using (1-(avg_rank-1))/(number_of_tools-1) so as to keep a score between 0 and 1. The best ranked tool is therefore the tool that has the greatest rank score.

#### Biological evaluation

Enrichment analysis was performed on the top 1000 DEGs identified using the scanpy.tl.rank_gene_groups function with the method parameter set to t-test. Gene set enrichment analysis was conducted using the gseapy package [48] version 1.1.3, specifically using the enrichr function. Gene sets were taken from the MSigDB resource [29], specifically the MSigDB_Hallmark_2020 collection, and from Gene Ontology [49], including GO_Biological_Process_2021, GO_Molecular_Function_2021, and GO_Cellular_Component_2021. For the H. Poly and the Nault dataset, organism variable was set to Mouse. Background was set to default. Enrichment terms were retained if the adjusted p-value was below 0.05. For each significant term, gene scores were calculated using scanpy.tl.score_genes, which computes the average expression of the selected gene set subtracted by the average expression of all other genes. AUCell scores were computed using the bioalpha package [50]. First, gene signatures for the interferon-alpha response were loaded for both the true perturbed cells and each predicted dataset. AUCell scores, representing pathway-level activity per cell, were then calculated and normalized for each dataset. The distributions of these scores were subsequently compared across tools using violin plots and statistically assessed with two-sided Kolmogorov–Smirnov tests.

#### Semantic similarity analysis

Semantic similarity was assessed using an ontology-based approach. We employed the Gene Ontology Biological Process (GO BP) ontology with human gene annotations from the org.Hs.eg.db database. Semantic relationships between GO terms were calculated using the GOSemSim package (version 2.24.0 [51]. Specifically, we used the Wang metric, which quantifies topological proximity between GO terms in the ontology graph and does not rely on corpus-derived information content. For each experiment, pairwise term–term similarity was computed between the predicted and perturbed GO term sets using this ontology-based metric, generating a full similarity matrix. The results were then summarized by calculating the mean of all pairwise similarity values, yielding a single representative measure of semantic relatedness for each experiment.

#### Construction of scArchon

scArchon was implemented in Snakemake (v8.28.0) and utilizes containerized environments available at https://hub.docker.com/u/hdsu. These Docker containers include all necessary software dependencies for the supported tools. The tools require CUDA versions ranging from 11.6 to 12.4; therefore, systems equipped with CUDA 12.4 or later are compatible with all components.

Execution of the pipeline also requires Singularity [52], tested on versions 3.6 and 4.1, with newer versions expected to be compatible. On average, each image occupies approximately 6.5 GB of disk space. Running for example four tools thus requires a minimum of ~ 30 GB to accommodate both images and output files. Once downloaded, the Singularity images are cached locally and reused, so subsequent runs do not incur additional download time.

Tool runtimes vary considerably. scPreGAN, scDisInFact, scPRAM, and scVIDR execute relatively quickly. CellOT is significantly slower due to the absence of GPU support and the fact that scGen has to be trained prior to extracting the latent space representation of the dataset. SCREEN and CPA also require extended runtime. Based on empirical results, it is recommended to exclude CellOT, SCREEN, and CPA from initial evaluations.

## Supplementary Information

Additional file 1: Supplementary figures. 

Additional file 2: Supplementary notes detailing the reproduction of available tools' results.

Additional file 3: Excel spreadsheet of all results presented in the manuscript. 

## Data Availability

The scArchon source code is publicly available on GitHub (https://github.com/hdsu-bioquant/scArchon) [53] and archived on Zenodo (https://doi.org/10.5281/zenodo.19820570) [54]. The code is available under MIT license. All the datasets used in this work are accessible on Zenodo (https://doi.org/10.5281/zenodo.19918636) [55].
