# Supplementary material for: scArchon: a scalable benchmarking framework for assessing single-cell perturbation models
Source: Genome Biol. 2026 May 12;27:162. doi: 10.1186/s13059-026-04104-z (PMC13162514; doi:10.1186/s13059-026-04104-z)
Supplement: Supplementary file 1 — Additional file 1: Supplementary figures. [file 13059_2026_4104_MOESM1_ESM.pdf]

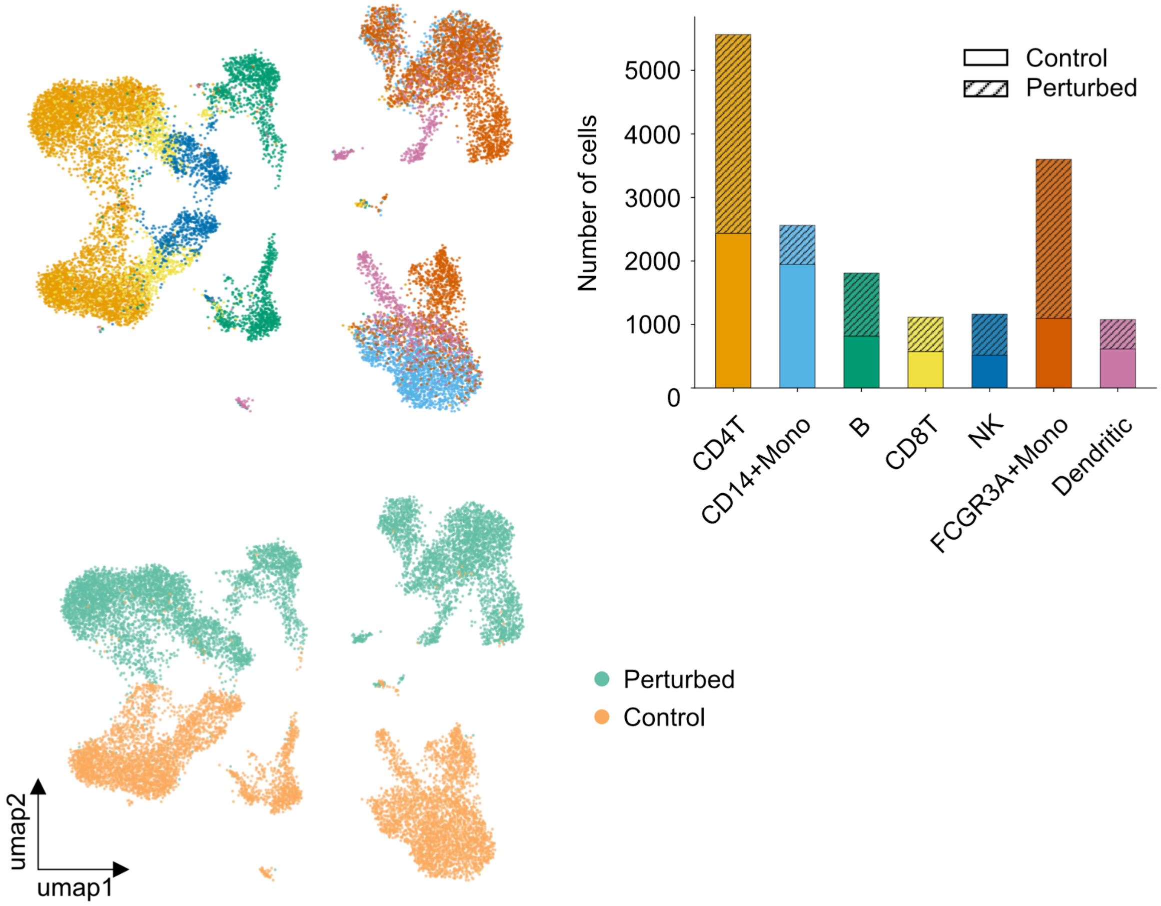

Fig. S1: Overview of the Kang dataset.
The top-left panel displays the full dataset, colored by cell type. The corresponding cell type distribution is summarized in the top-right bar plot. The bottom-left panel highlights the distribution of cells across the perturbed and control conditions. The dataset includes 16,893 cells, from which 8,886 cells in control and 8,007 cells in perturbed, and 6,998 genes.

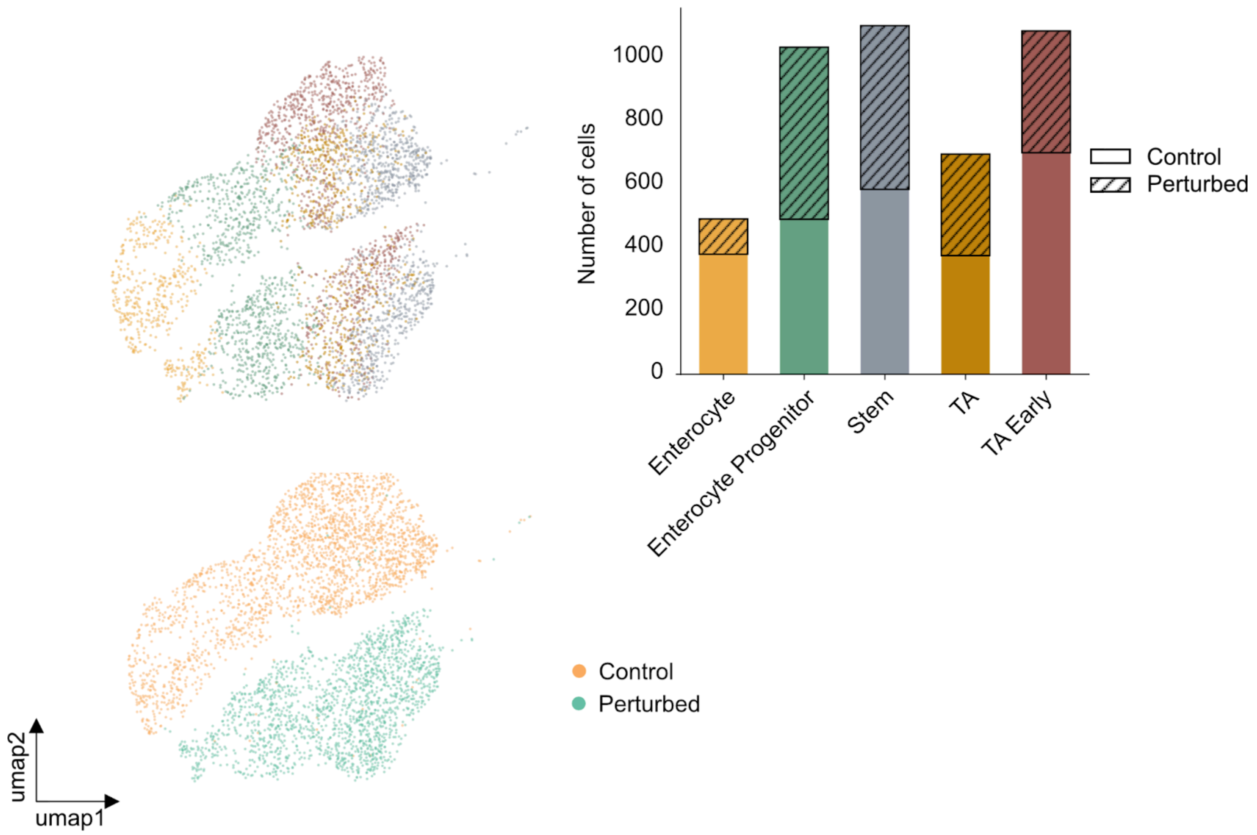

Fig. S2: Overview of the H. Poly dataset.
The top-left panel displays different cell types, colored according to the distribution shown in the top-right bar plot. The bottom-left panel illustrates the separation between control and perturbed cells. The dataset contains 4,192 cells and 7,000 genes, including 2,406 control cells and 1,786 perturbed cells.

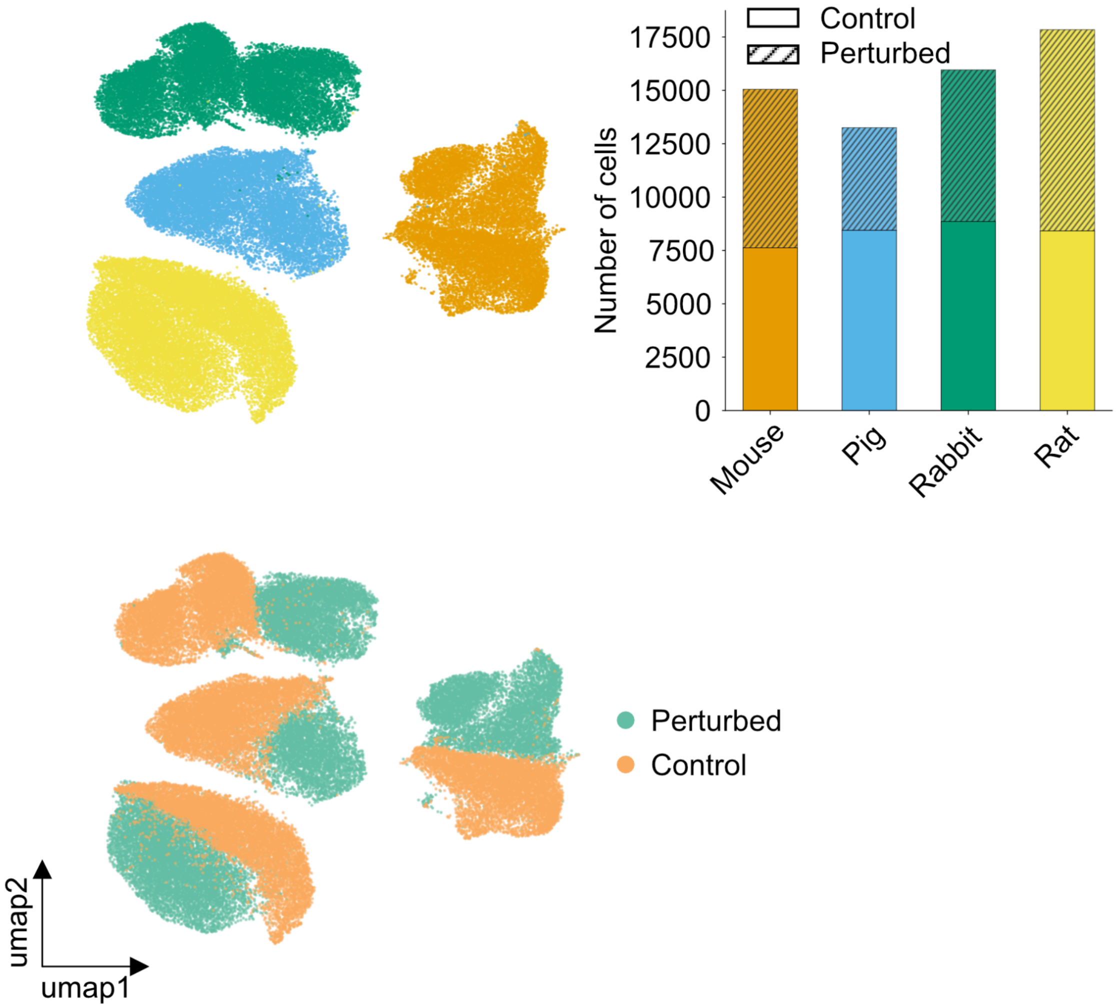

Fig. S3: Overview of the Species dataset.
The top-left panel displays different cell types, colored according to the distribution shown in the top-right bar plot. The bottom-left panel illustrates the separation between control and perturbed cells. The dataset consists of 62,114 cells and 6,619 genes, including 33,350 control cells and 28,764 perturbed cells.

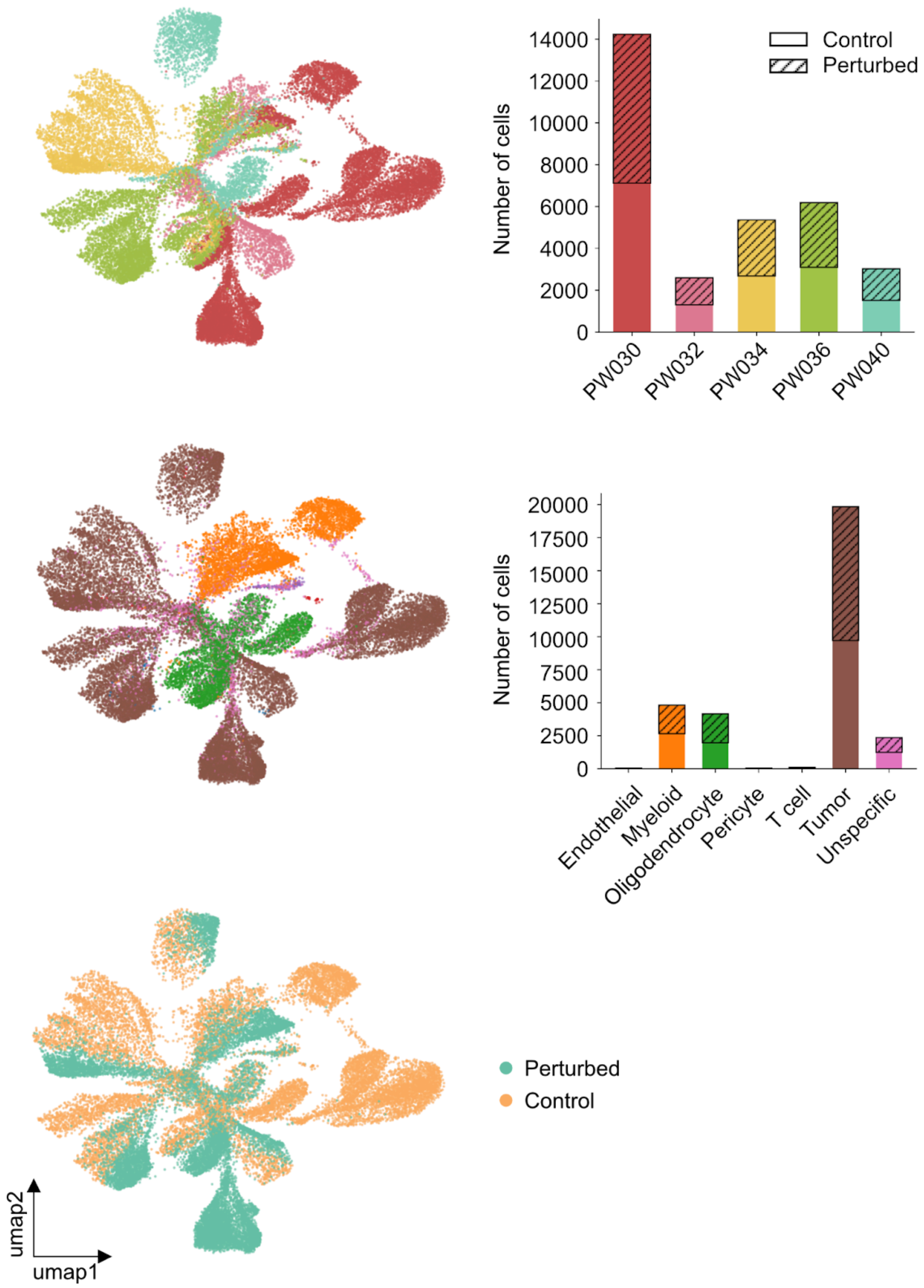

Fig. S4: Overview of the Glioblastoma dataset.
The top-left panel shows different cell types, colored as in the bar plot on the top-right. The bottom-left panel displays control and perturbed cells. The dataset comprises 31,414 cells and 7,000 genes, with patient and cell type annotations. It includes 15,707 control cells and 15,707 perturbed cells.

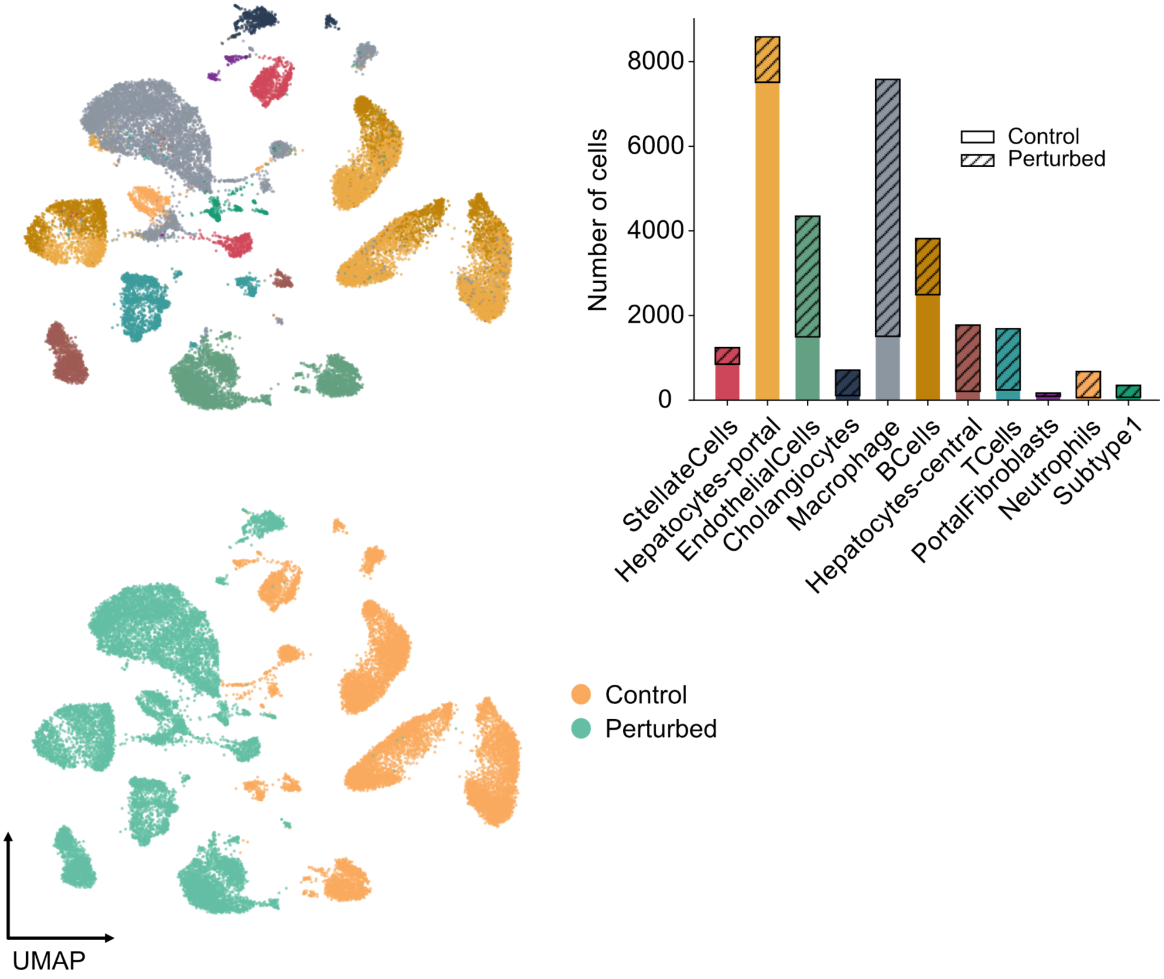

Fig. S5: Overview of the Nault dataset.
The top-left panel shows different cell types, colored as in the bar plot on the top-right. The bottom-left panel displays control and perturbed cells. The dataset comprises 30,927 cells and 6,999 genes. It includes 14,6333 control cells and 16,294 perturbed cells.

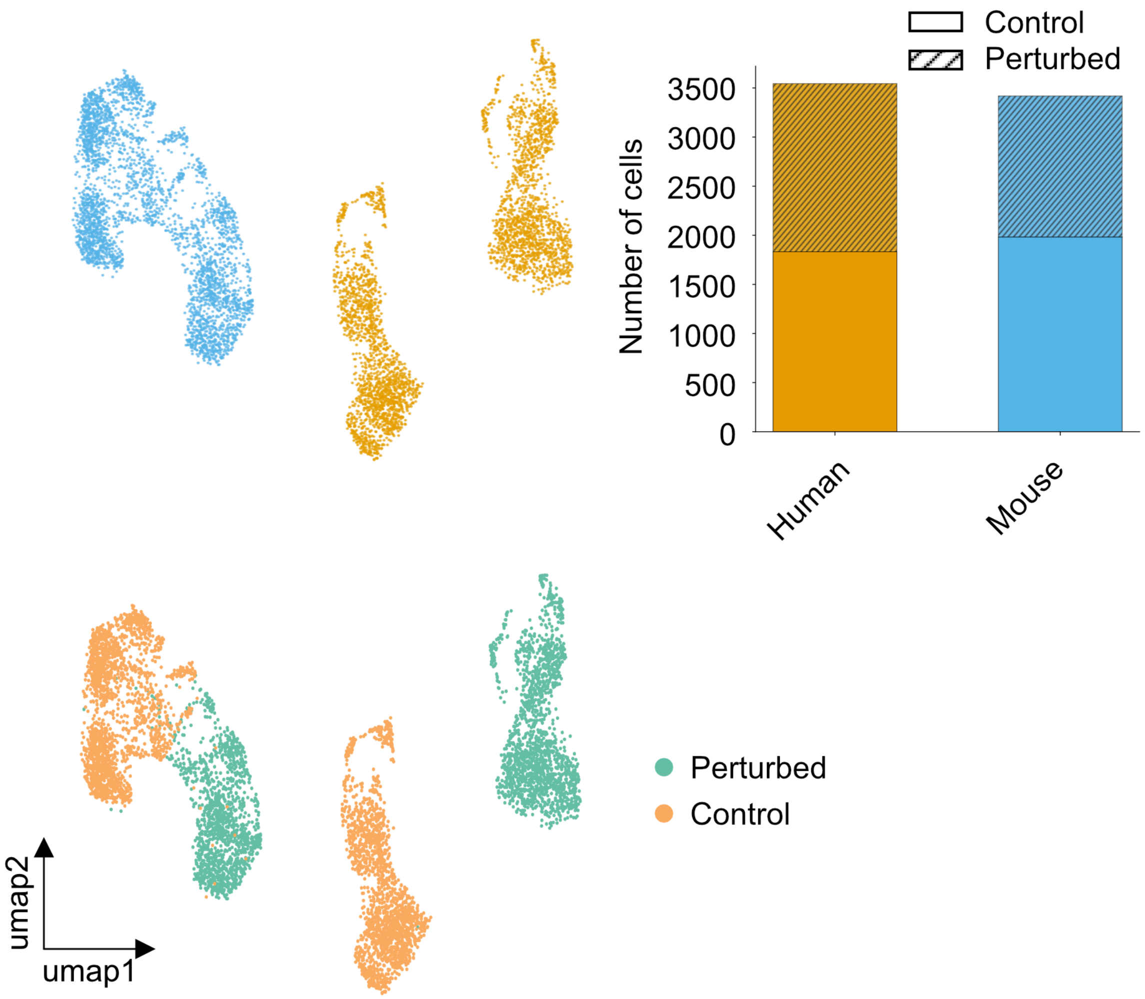

Fig. S6: Overview of the Interferon alpha dataset.
The top-left panel shows cells pertaining to the different species, colored as in the bar plot on the top-right. The bottom-left panel displays control and perturbed cells. The dataset contains T-cells only. The dataset comprises 6,964 cells and 12,542 genes. It includes 3,815 control cells and 3,149 perturbed cells.

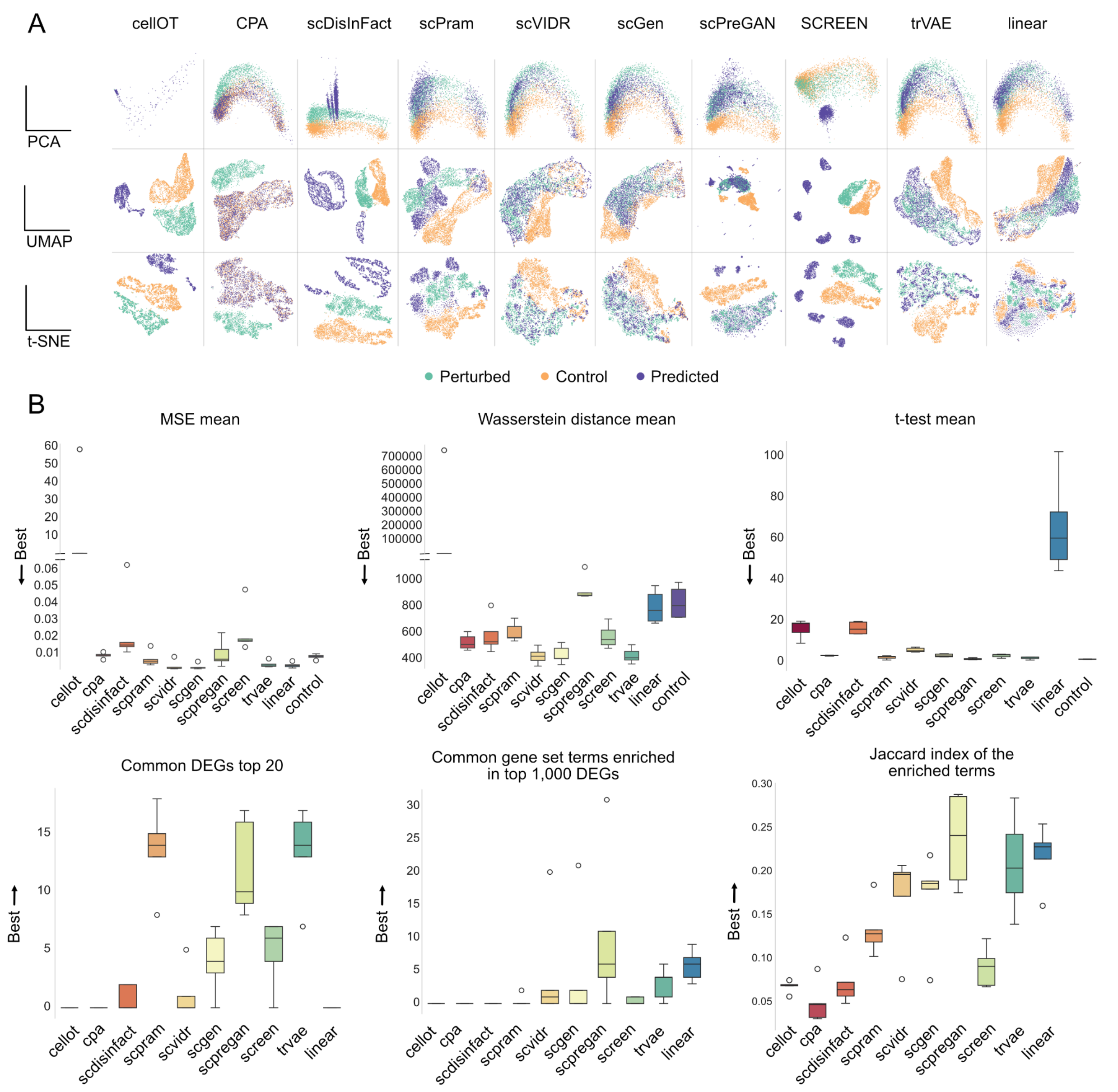

Fig. S7: Results on the H. Poly dataset.

A. Dimensionality reduction plot showing the aggregated outputs from all experiments conducted on the dataset.

B. Evaluation metrics including MSE, Wasserstein distance, t-test, overlap of top 20 differentially expressed genes (DEGs), and overlap of top 1,000 DEGs used for enrichment analysis.

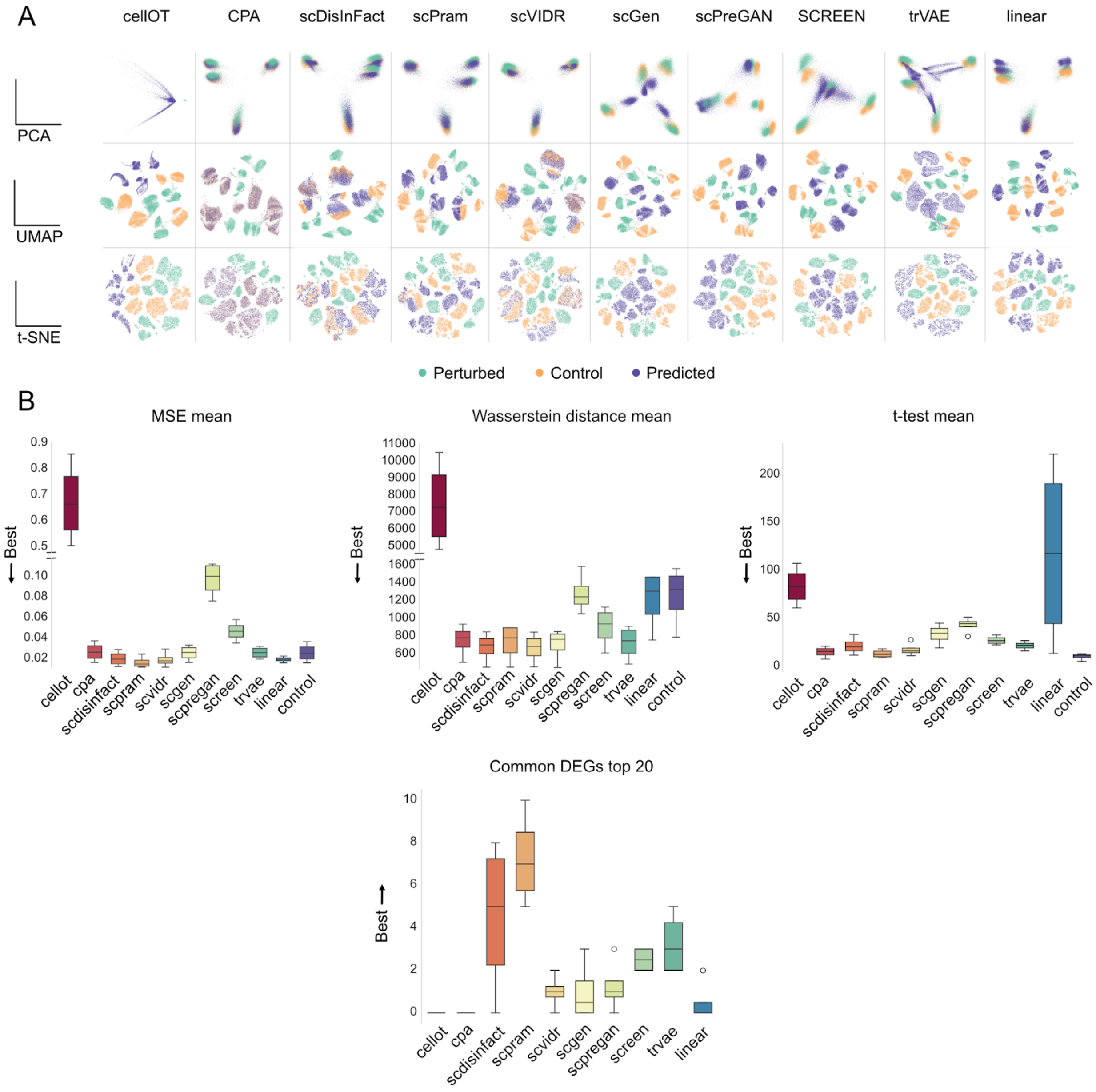

Fig. S8: Results on the Species dataset.

A. Dimensionality reduction plot showing the aggregated outputs from all experiments conducted on the dataset.

B. Evaluation metrics including MSE, Wasserstein distance, t-test and overlap of top 20 differentially expressed genes (DEGs).

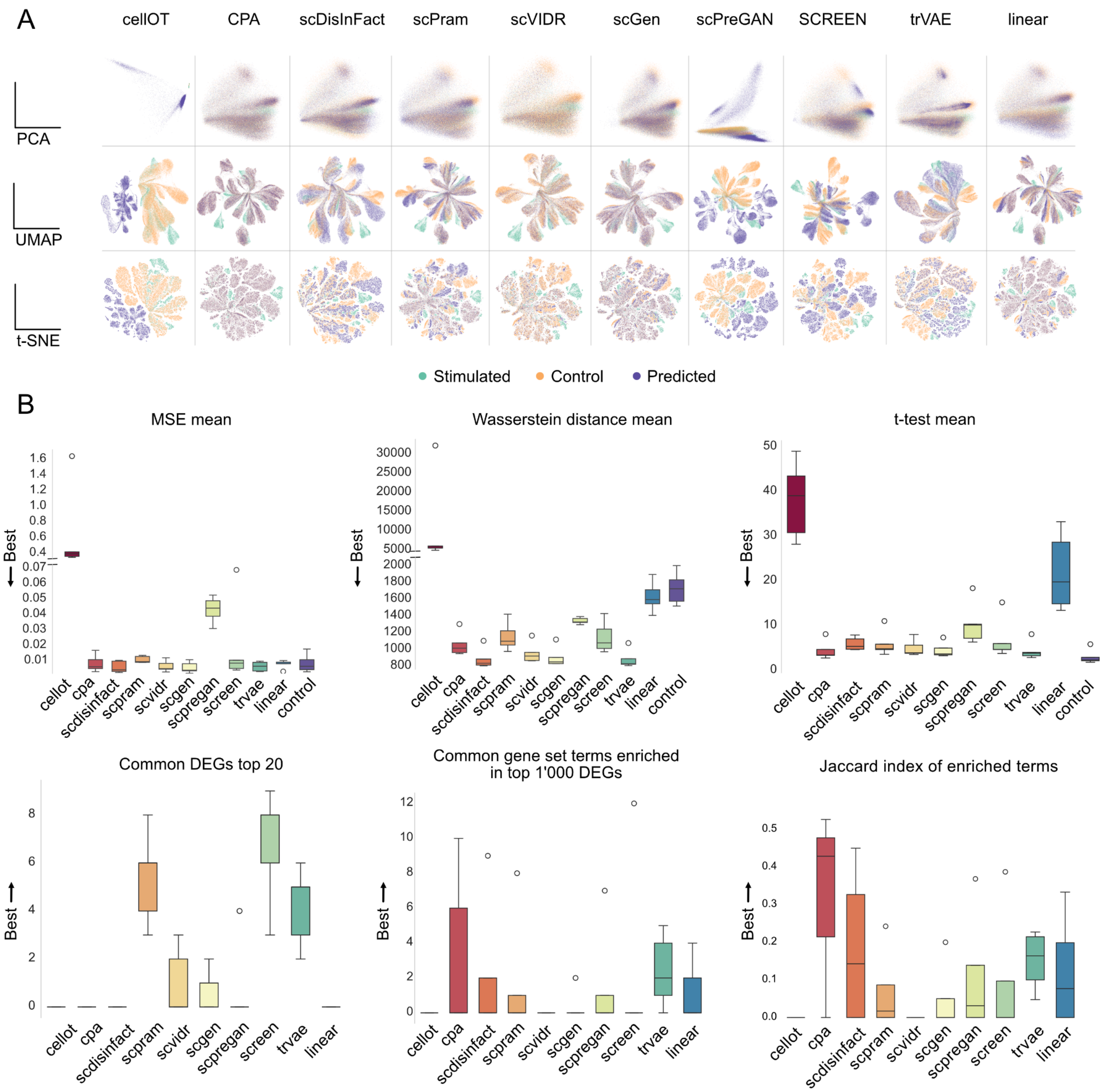

Fig. S9: Results on the Glioblastoma dataset.

A. Dimensionality reduction plot showing the aggregated outputs from all experiments conducted on the dataset.

B. Evaluation metrics including MSE, Wasserstein distance, t-test, overlap of top 20 differentially expressed genes (DEGs), and overlap of top 1,000 DEGs used for enrichment analysis.

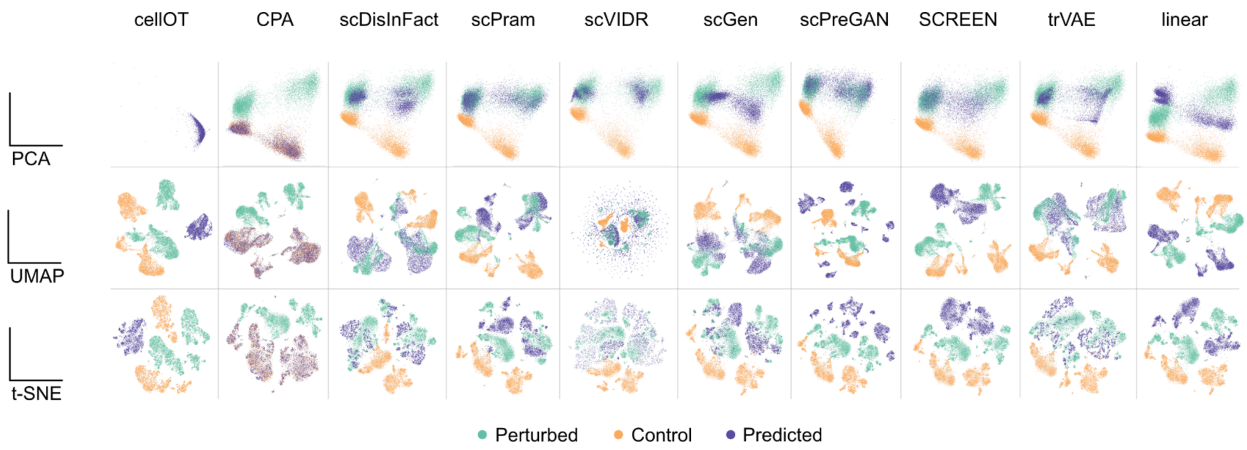

Fig. S10: Dimensionality reduction plot showing the aggregated outputs from all experiments conducted on the Kang dataset.

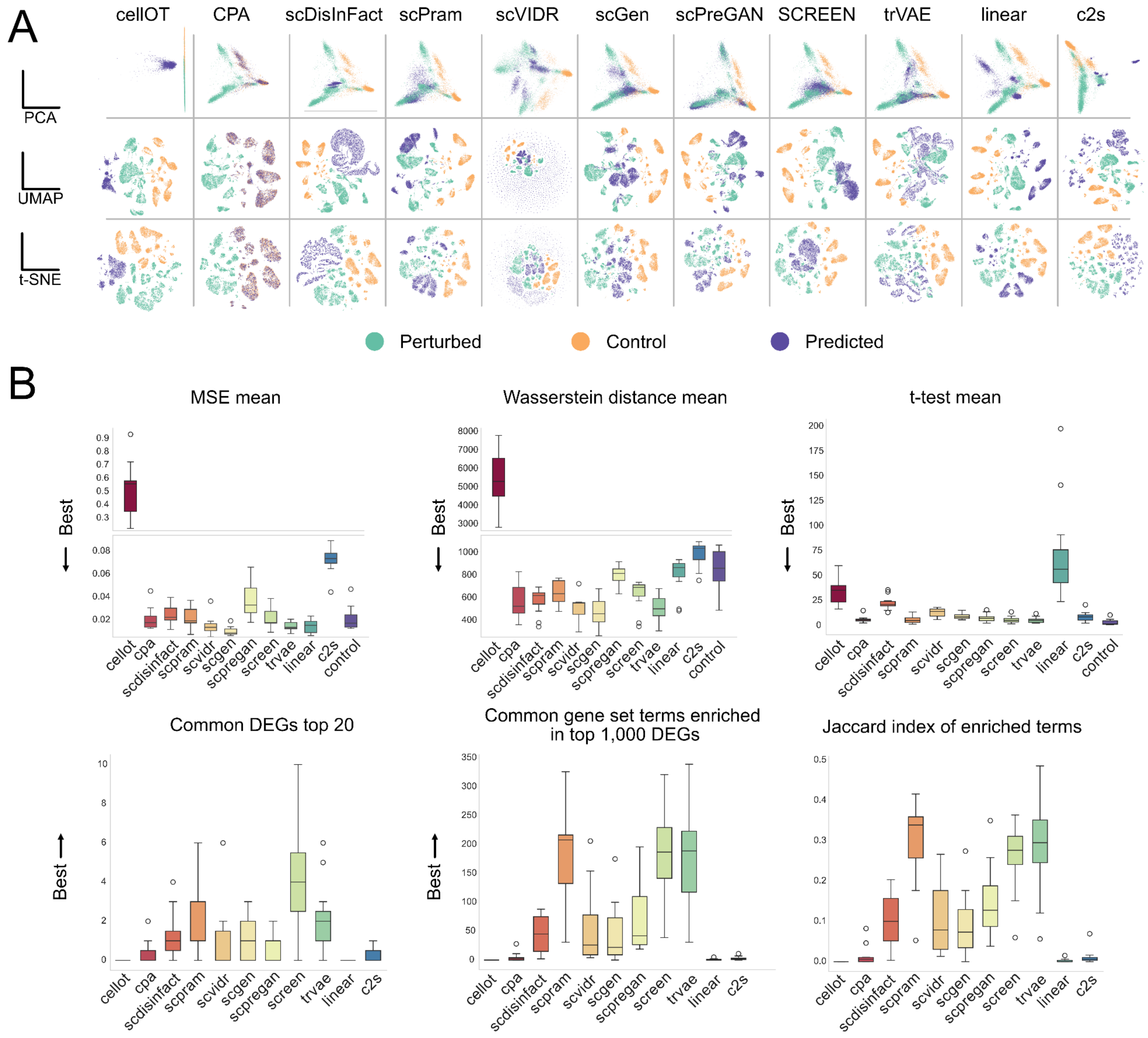

Fig. S11: Results on the Nault dataset.

A. Dimensionality reduction plot showing the aggregated outputs from all experiments conducted on the dataset.

B. Evaluation metrics including MSE, Wasserstein distance, t-test, overlap of top 20 differentially expressed genes (DEGs), and overlap of top 1,000 DEGs used for enrichment analysis.

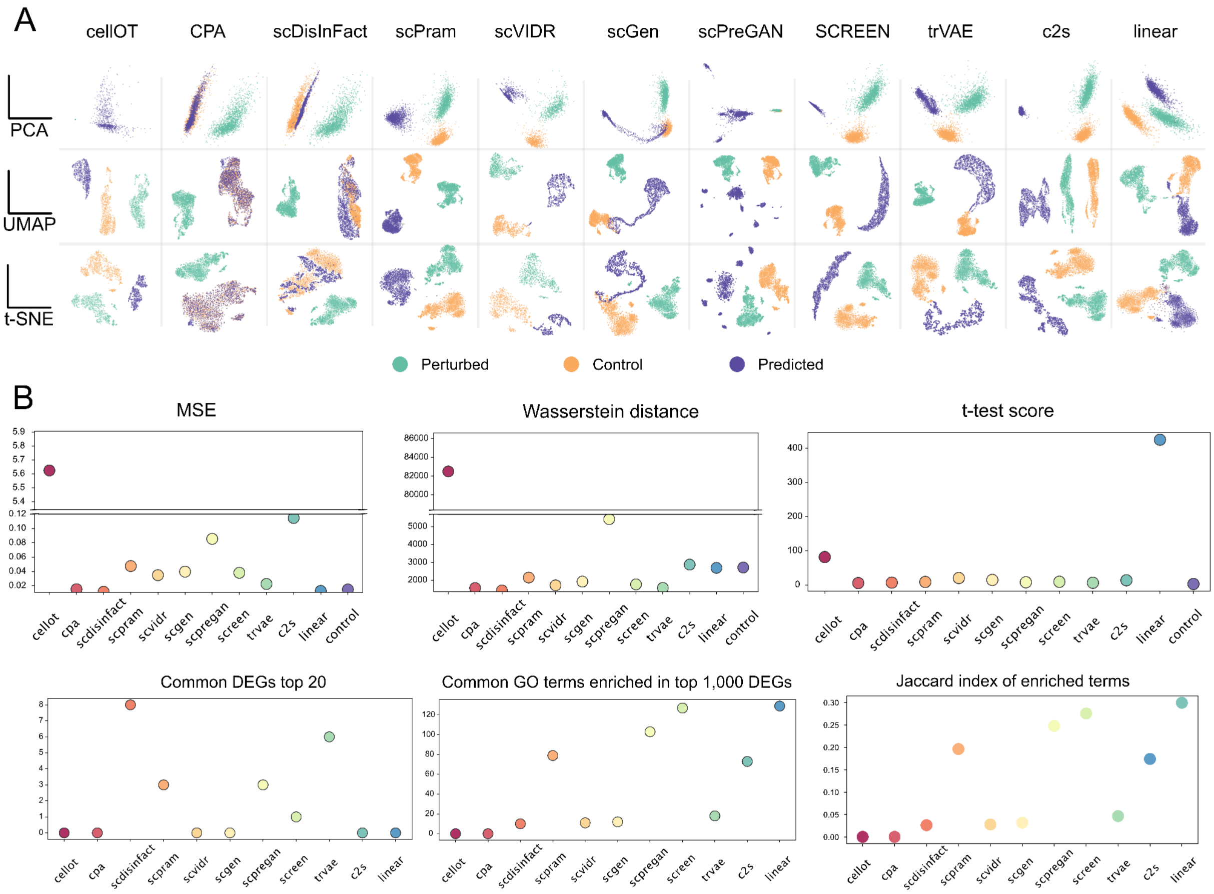

Fig. S12: Results on the Interferon alpha dataset.

A. Dimensionality reduction plot showing the aggregated outputs from all experiments conducted on the dataset.

B. Evaluation metrics including MSE, Wasserstein distance, t-test, overlap of top 20 differentially expressed genes (DEGs), and overlap of top 1,000 DEGs used for enrichment analysis.

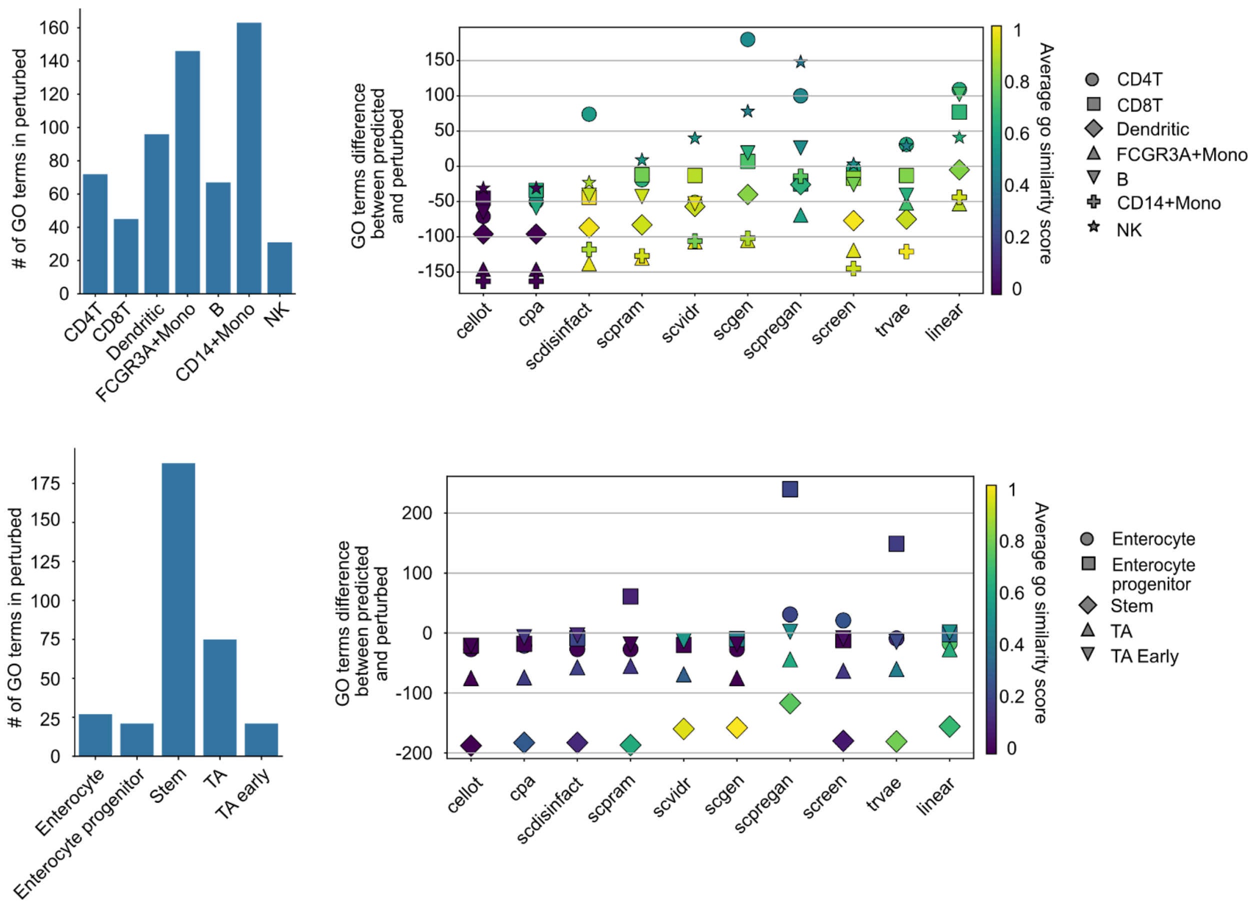

Fig. S13: Similarity scores for the two datasets with enriched GO terms. The top panel shows scores for the Kang dataset and the bottom panel for the H. Poly dataset.

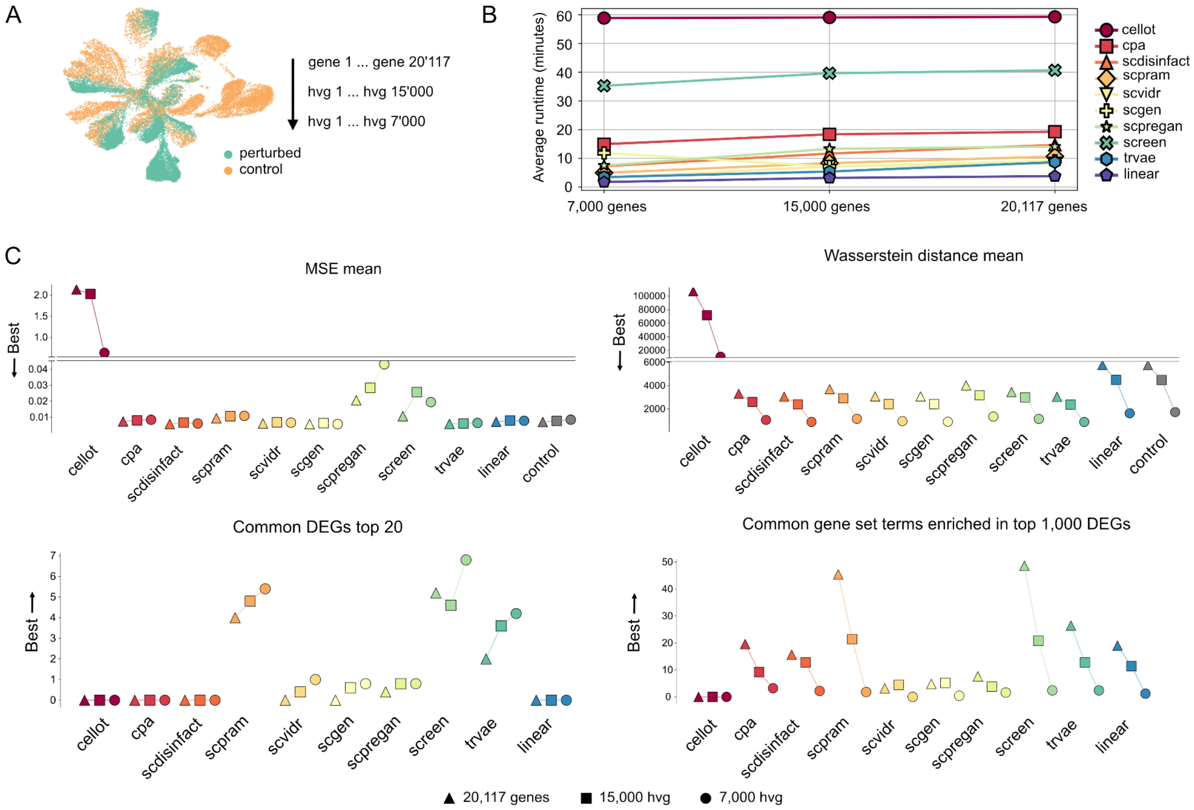

Fig. S14: Gene ablation experiment.

A. Schematic of the experiment. Starting with the Glioblastoma dataset, we progressively reduce the number of input genes—from the full set to the top 15,000 highly variable genes (HVGs), and then to the top 7,000 HVGs.

B. Runtime comparison of the different tools across datasets with varying gene counts.

C. Metric trends as gene count decreases. Top left: Mean Squared Error (MSE); top right: Wasserstein distance; bottom left: number of differentially expressed genes (DEGs) shared between prediction and reference; bottom right: number of shared enriched gene sets.

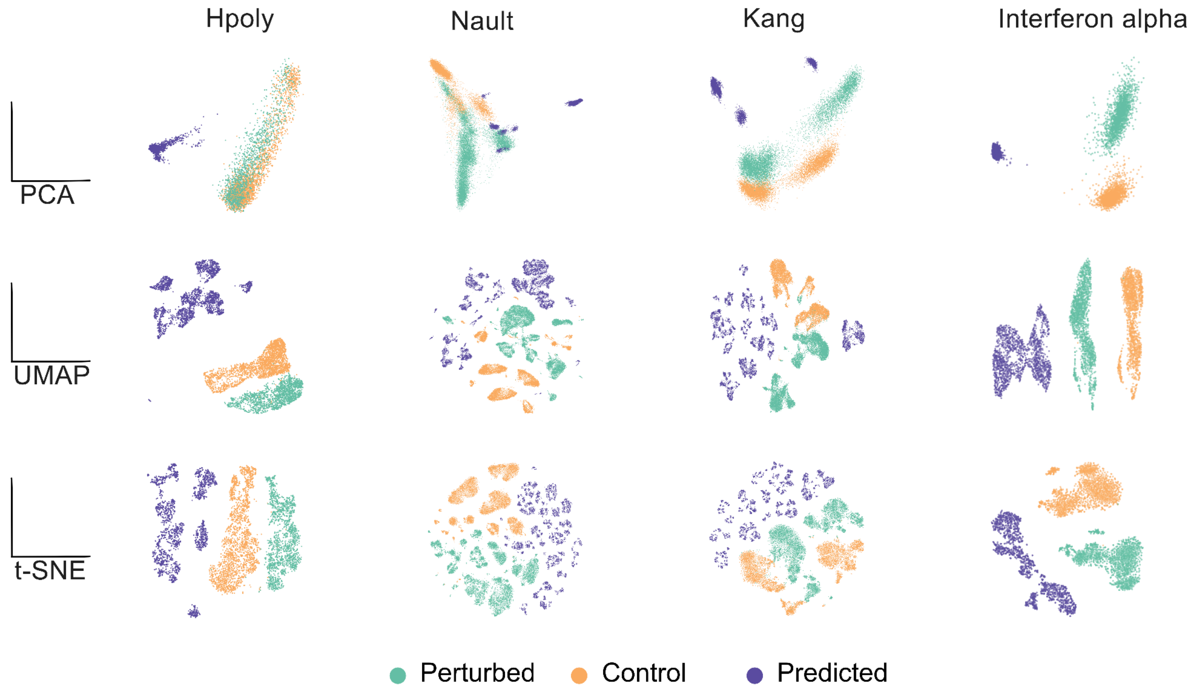

Fig. S15: c2s results across all evaluated datasets.
Shown are the low-dimensional embeddings of the c2s predictions for each dataset on which the method was applied, illustrating its behavior and output structure across diverse perturbation settings.

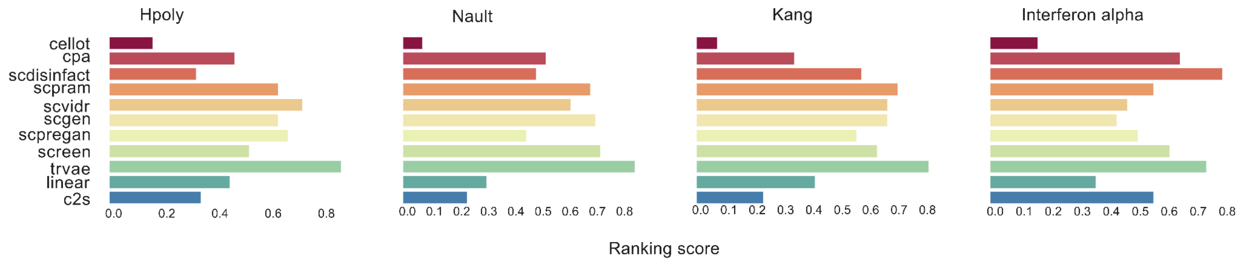

Fig. S16: Performance ranking of c2s relative to other tools on the datasets where it could be applied.

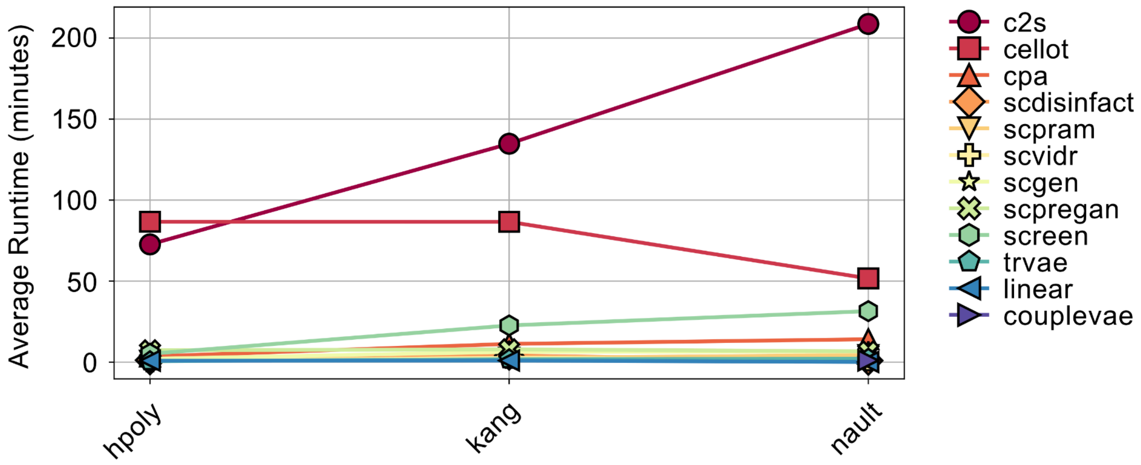

Fig. S17: Runtime comparison of all tools on the datasets where c2s could be executed. This figure reports the computational runtime of each method on the subset of datasets for which c2s successfully ran.

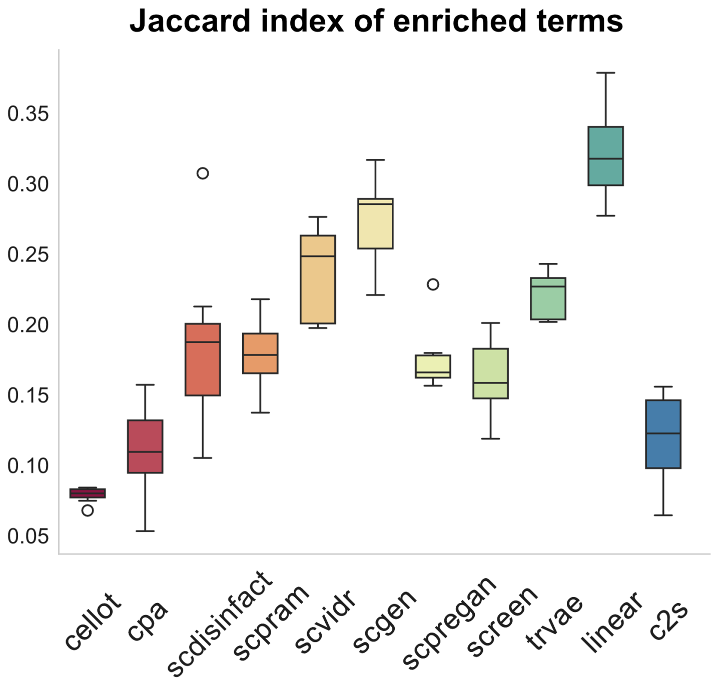

Fig. S18: Jaccard index of enriched terms for the Kang dataset

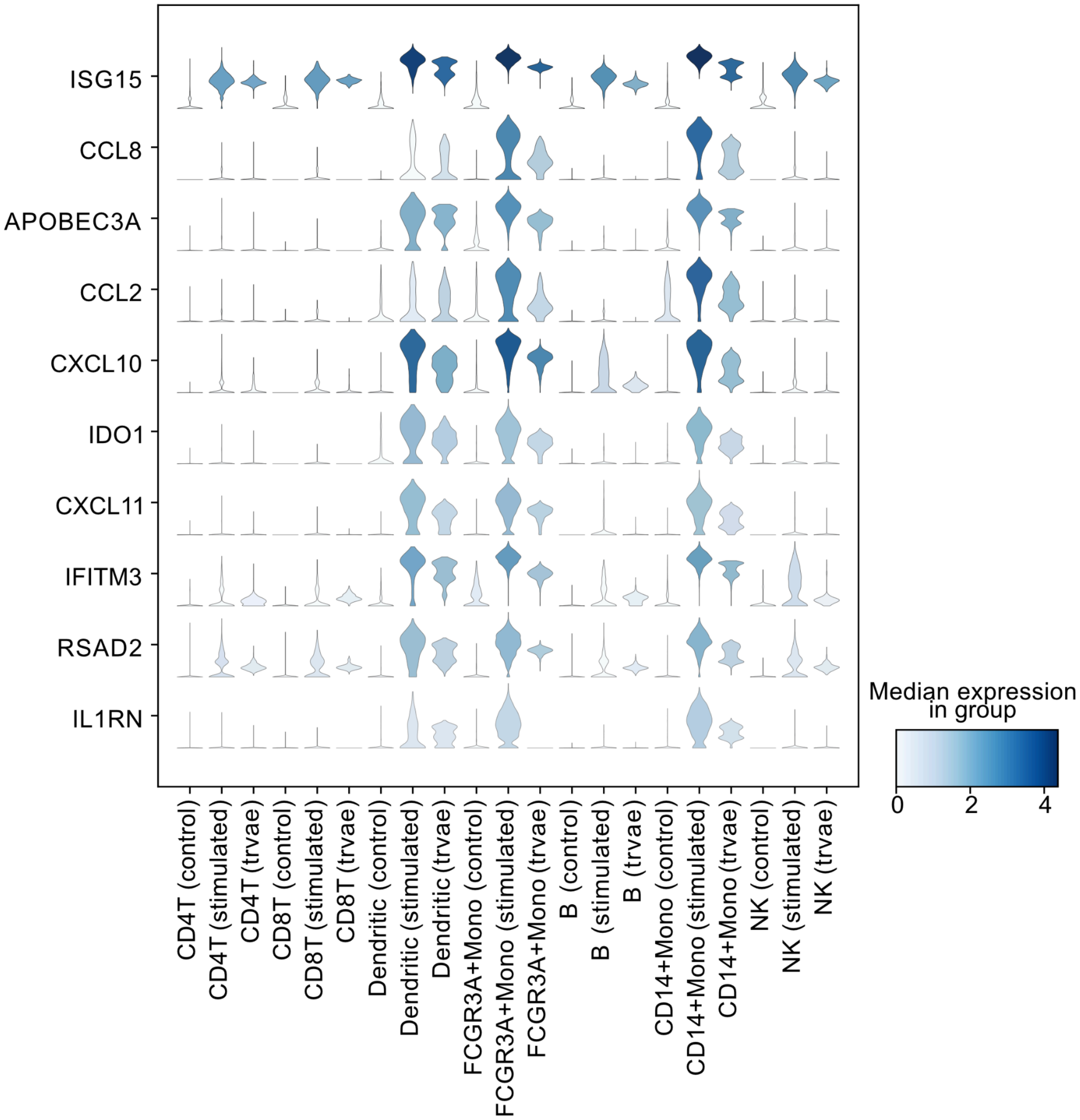

Fig. S19: Comparison of gene expression distributions in control, stimulated, and trVAE-predicted cells for a selected set of genes responsive to interferon treatment. Expression levels are shown separately for each cell type. Computed on the Kang dataset.

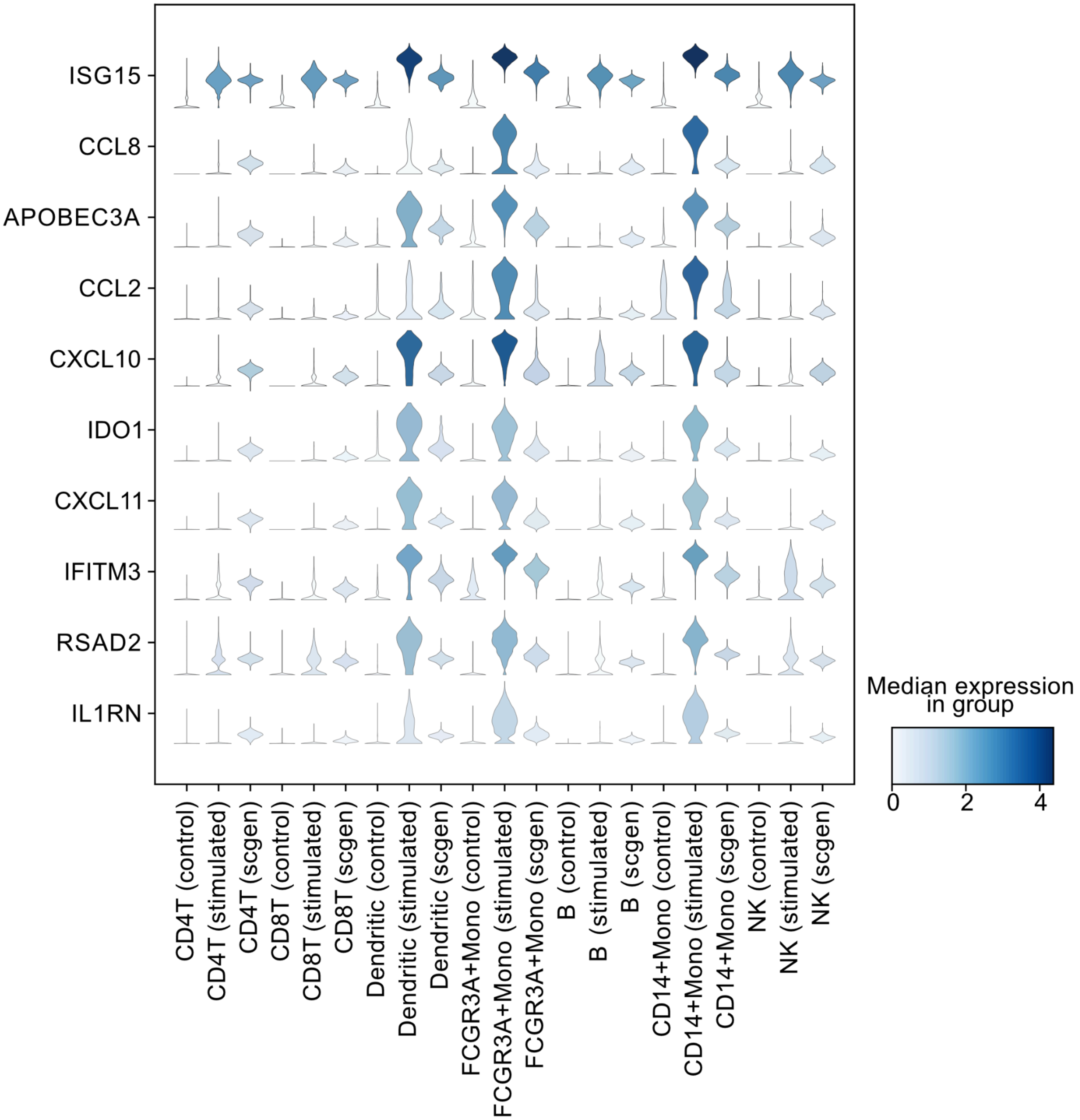

Fig. S20: Comparison of gene expression distributions in control, stimulated, and scGen-predicted cells for a selected set of genes responsive to interferon treatment. Expression levels are shown separately for each cell type. Computed on the Kang dataset.

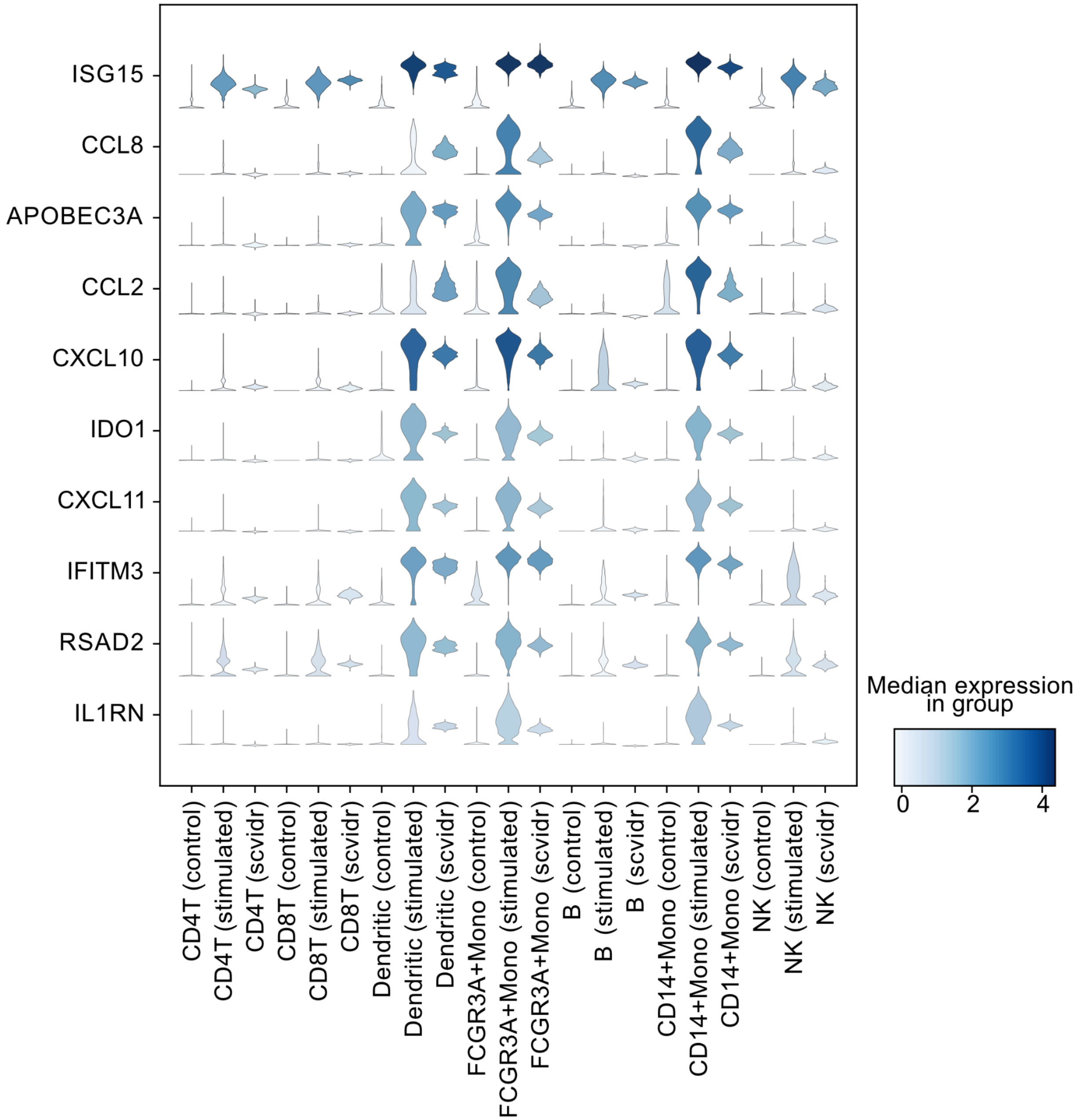

Fig. S21: Comparison of gene expression distributions in control, stimulated, and scVIDR-predicted cells for a selected set of genes responsive to interferon treatment. Expression levels are shown separately for each cell type. Computed on the Kang dataset.

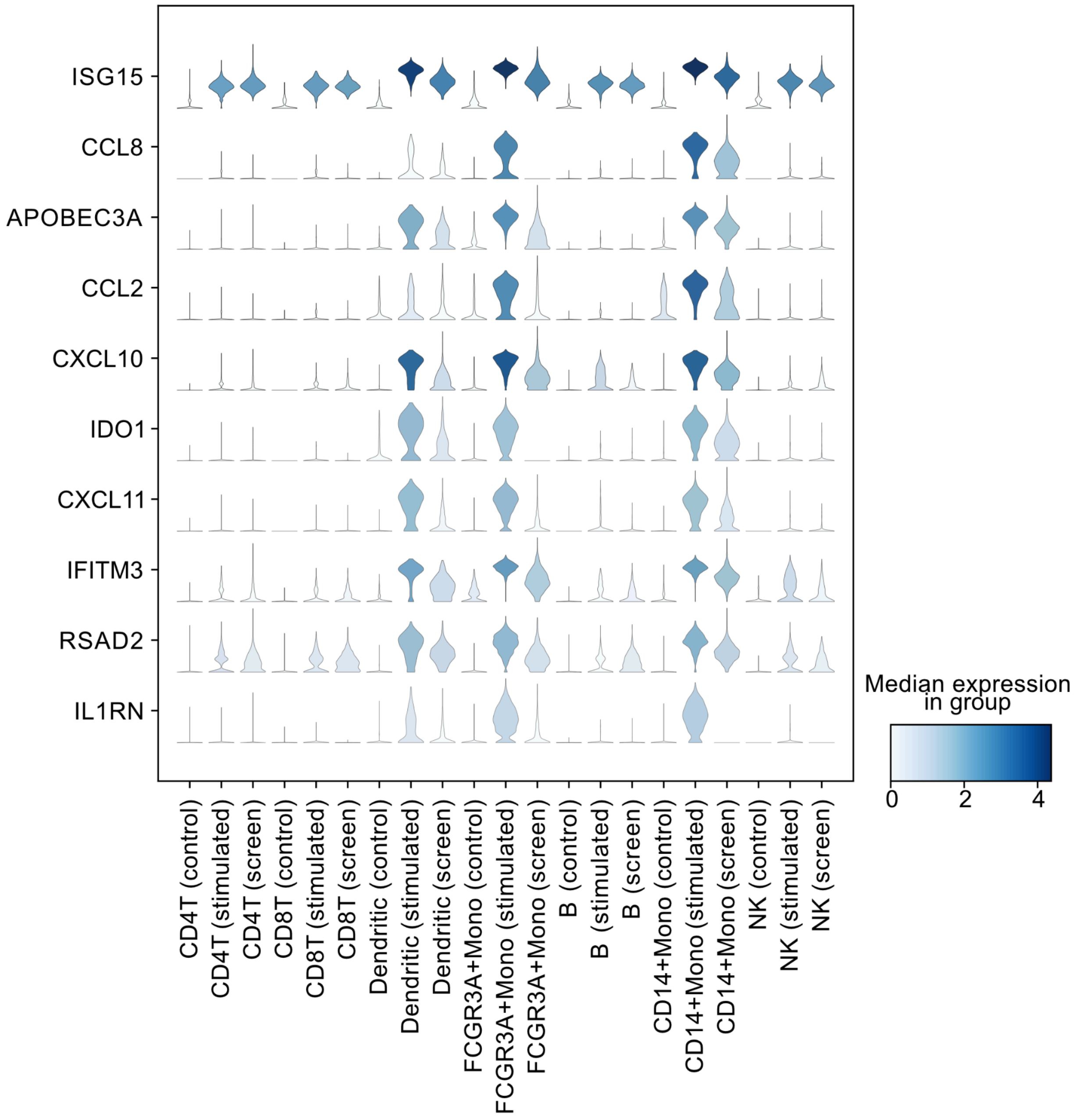

Fig. S22: Comparison of gene expression distributions in control, stimulated, and SCREEN-predicted cells for a selected set of genes responsive to interferon treatment. Expression levels are shown separately for each cell type. Computed on the Kang dataset.

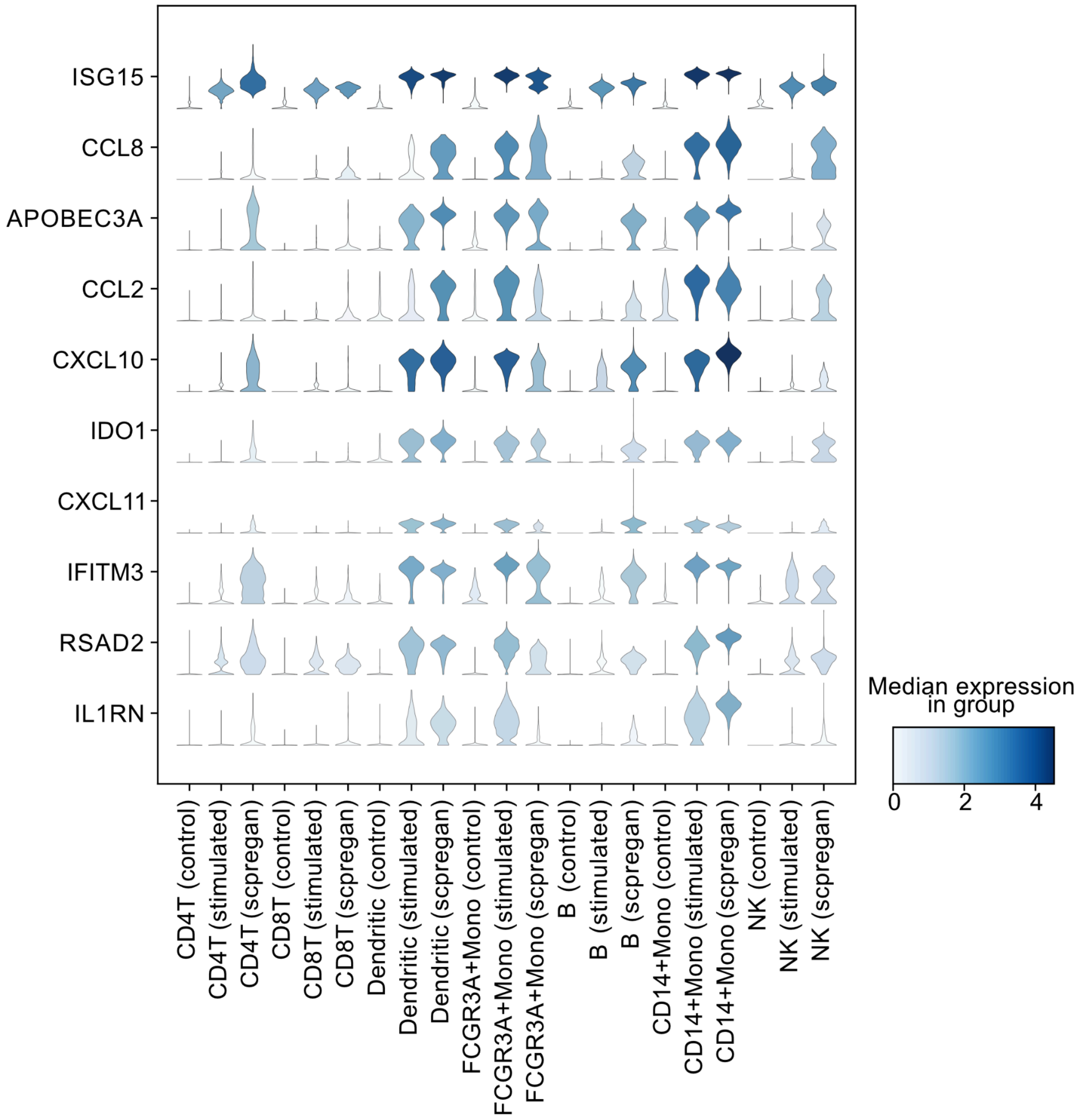

Fig. S23: Comparison of gene expression distributions in control, stimulated, and scPreGAN-predicted cells for a selected set of genes responsive to interferon treatment. Expression levels are shown separately for each cell type. Computed on the Kang dataset.

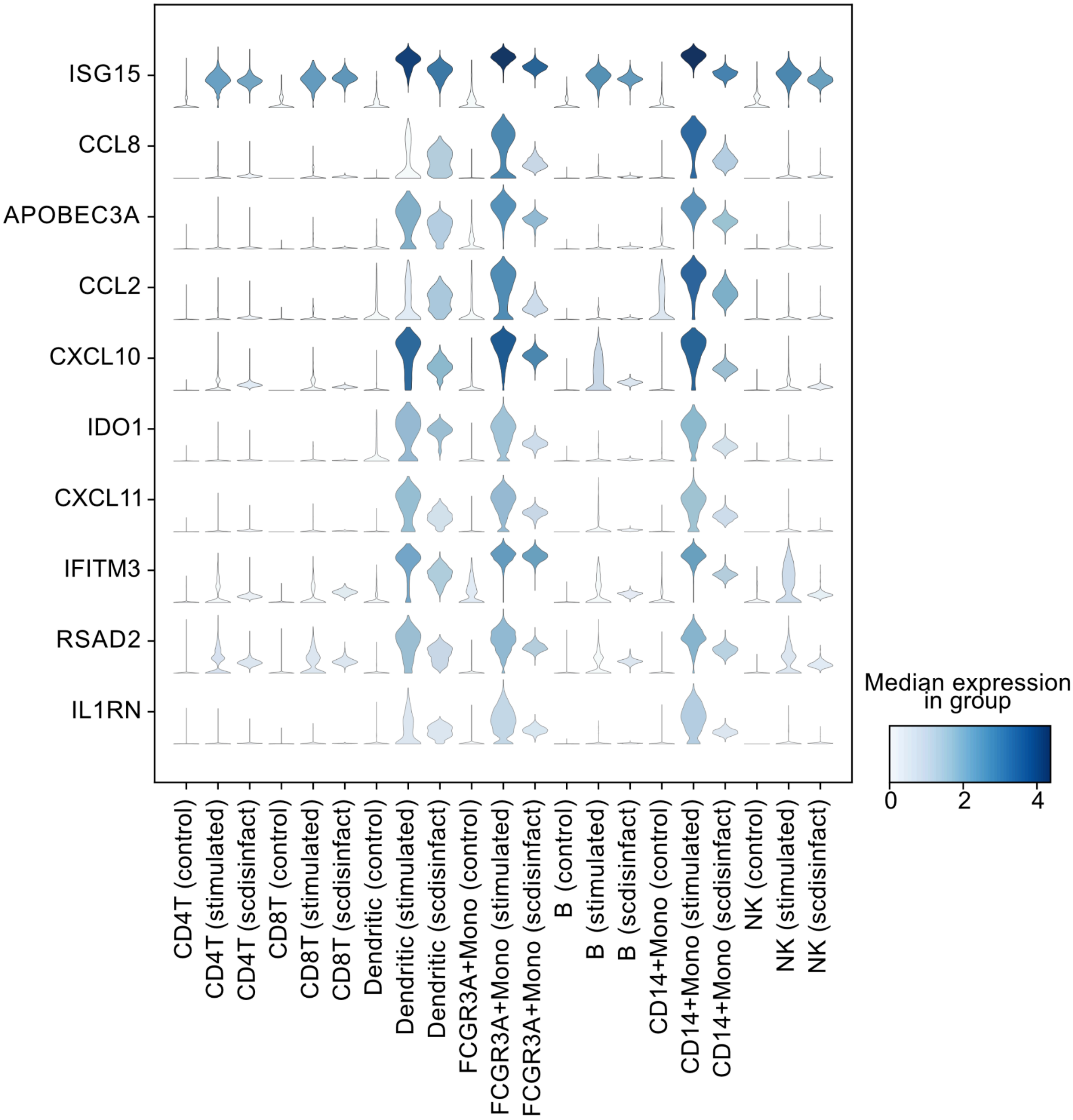

Fig. S24: Comparison of gene expression distributions in control, stimulated, and scDisInFact-predicted cells for a selected set of genes responsive to interferon treatment. Expression levels are shown separately for each cell type. Computed on the Kang dataset.

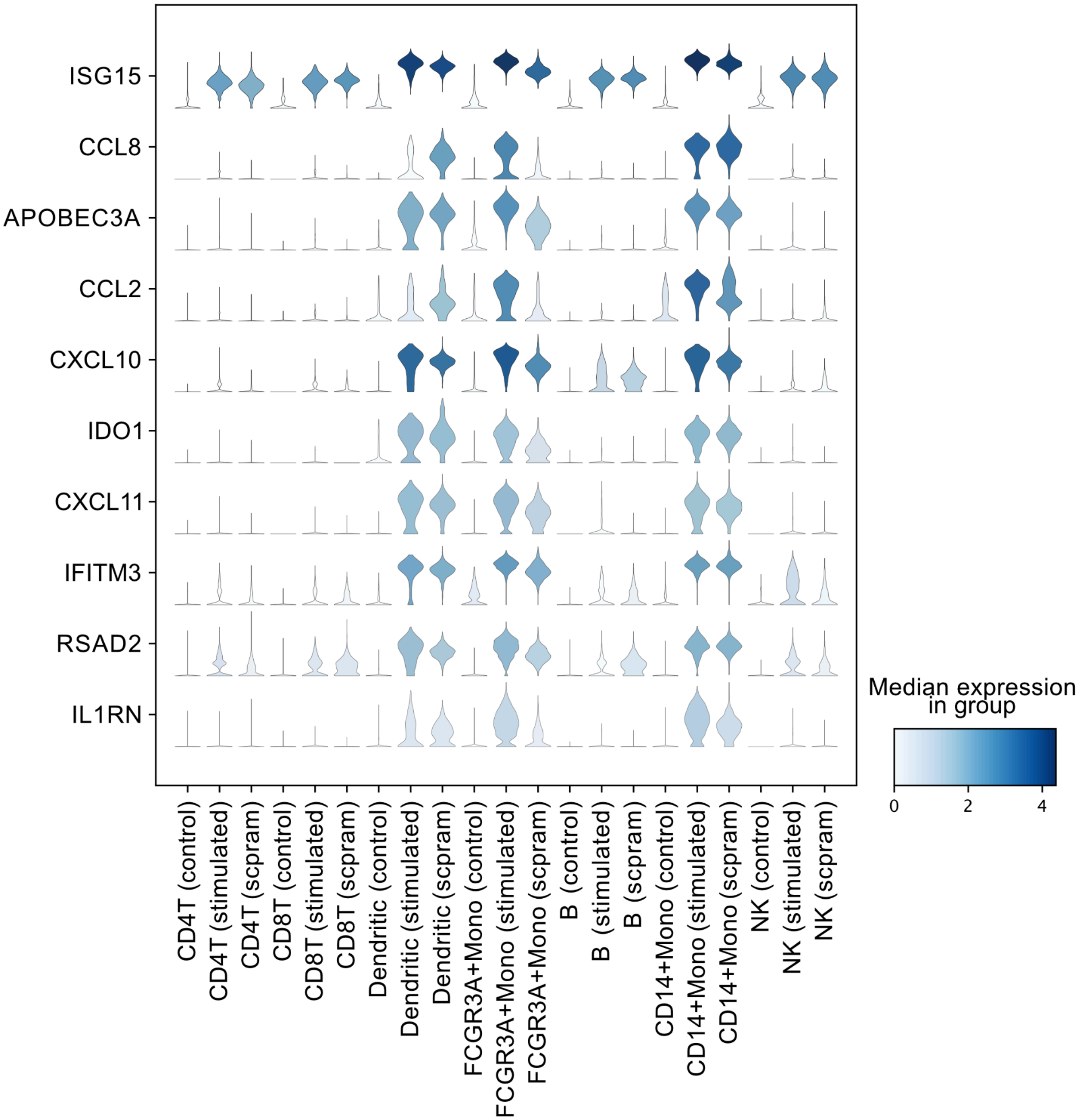

Fig. S25: Comparison of gene expression distributions in control, stimulated, and scPram-predicted cells for a selected set of genes responsive to interferon treatment. Expression levels are shown separately for each cell type. Computed on the Kang dataset.

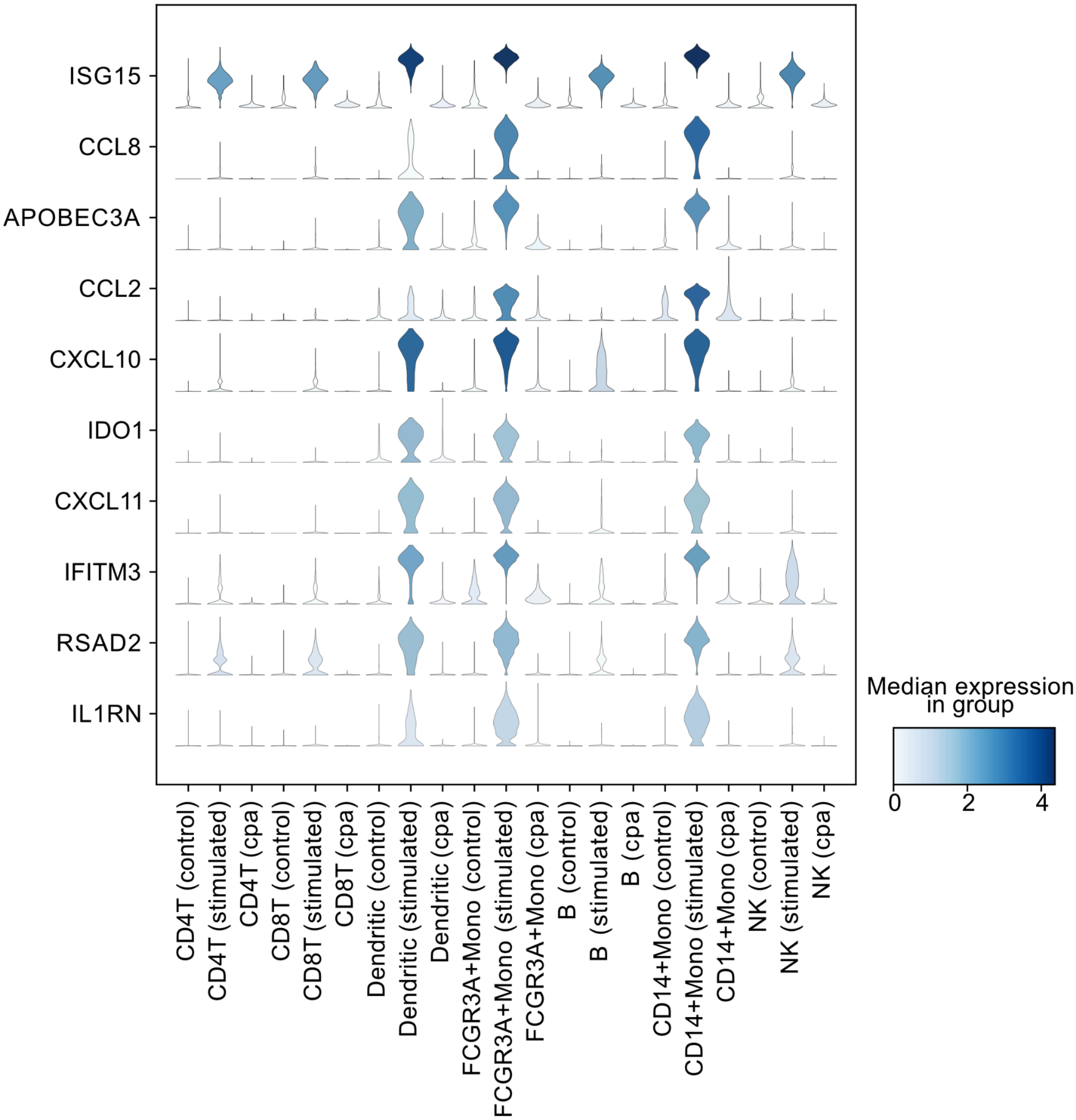

Fig. S26: Comparison of gene expression distributions in control, stimulated, and CPA-predicted cells for a selected set of genes responsive to interferon treatment. Expression levels are shown separately for each cell type. Computed on the Kang dataset.

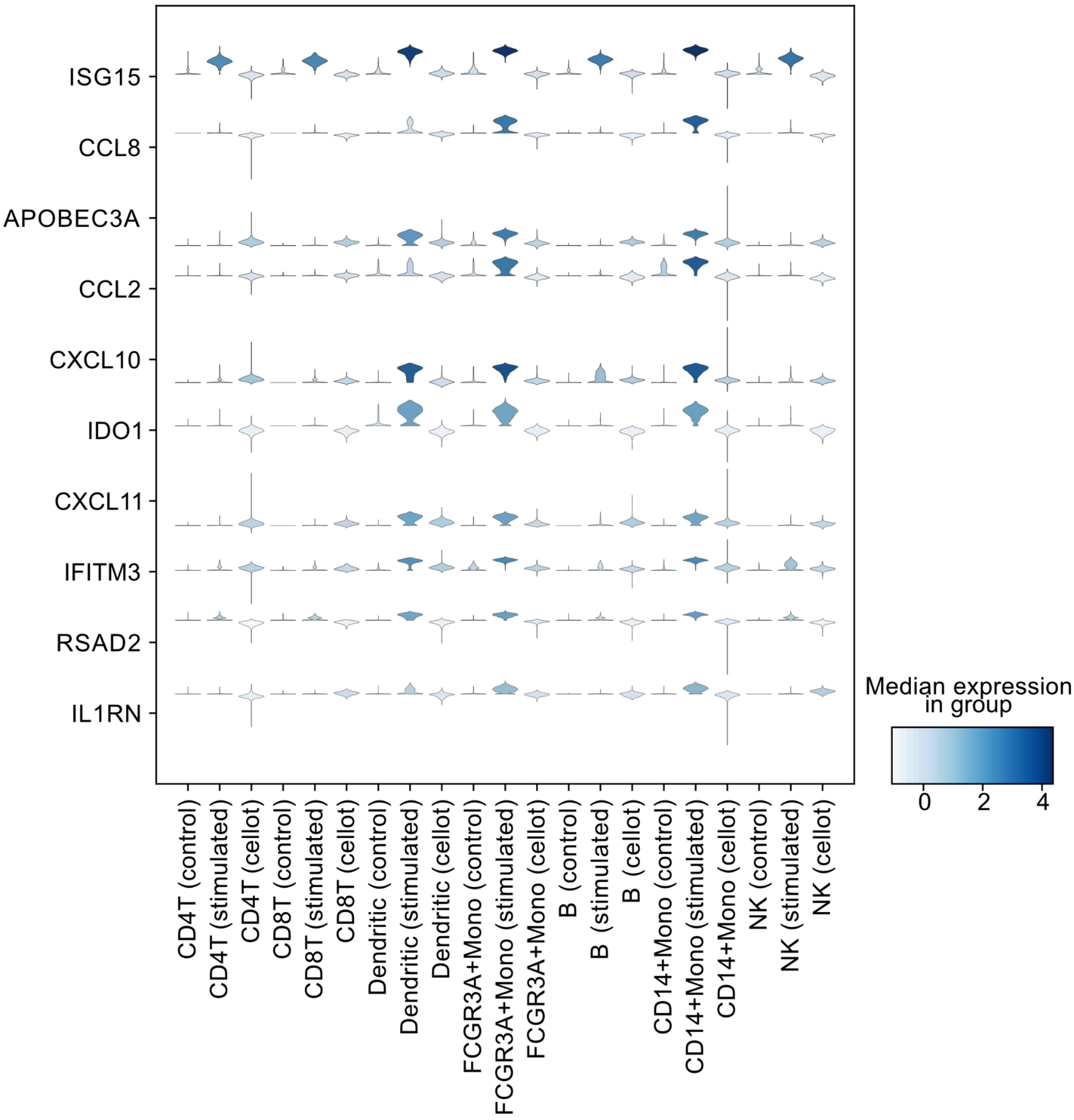

Fig. S27: Comparison of gene expression distributions in control, stimulated, and cellOT-predicted cells for a selected set of genes responsive to interferon treatment. Expression levels are shown separately for each cell type. Computed on the Kang dataset.

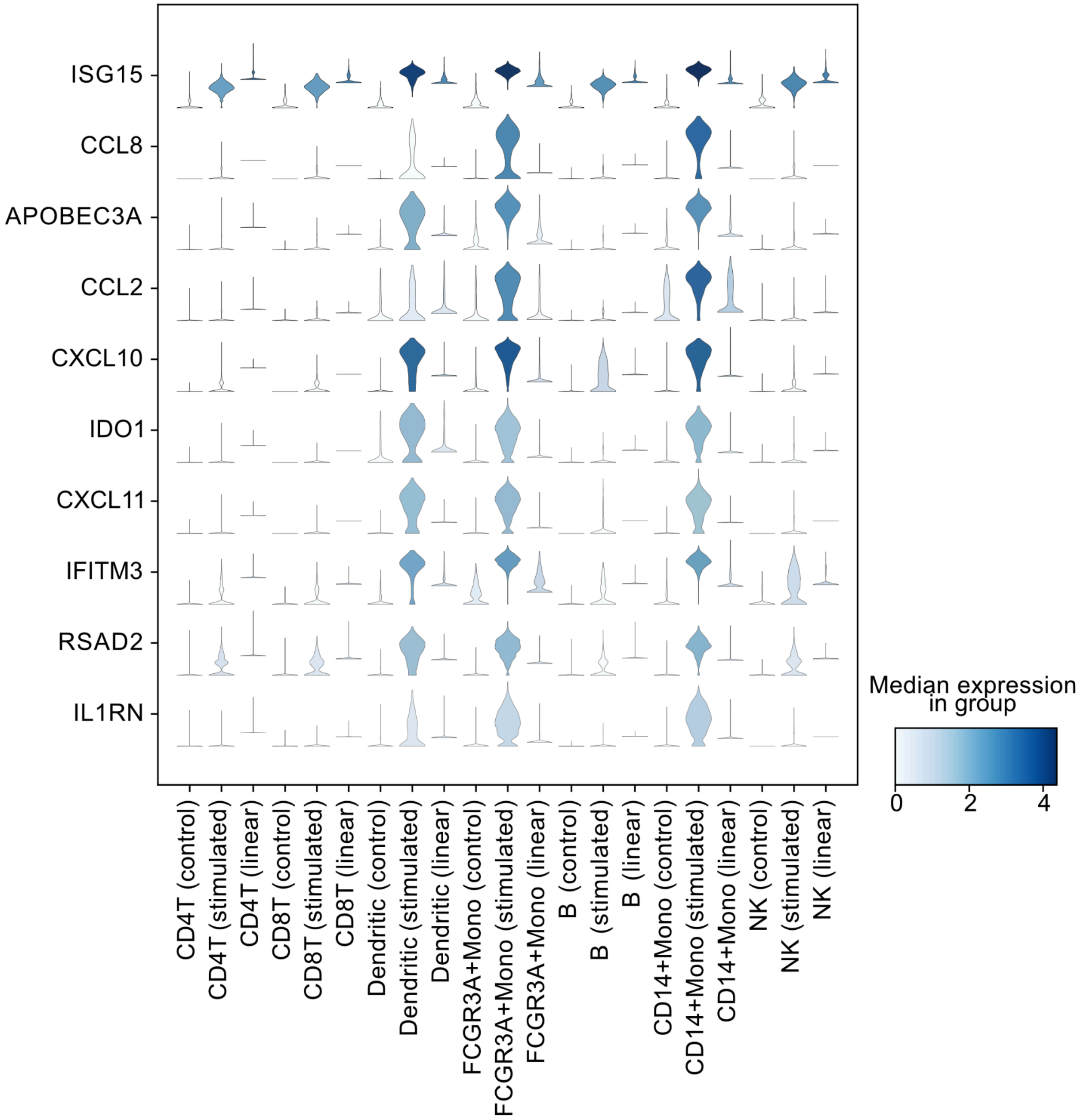

Fig. S28: Comparison of gene expression distributions in control, stimulated, and predicted cells from the linear model for a selected set of genes responsive to interferon treatment. Expression levels are shown separately for each cell type. Computed on the Kang dataset.
